# AAV-mediated hepatic expression of SLC30A10 and the Thr95Ile variant attenuates manganese excess and other phenotypes in Slc30a10-deficient mice

**DOI:** 10.1016/j.jbc.2024.105732

**Published:** 2024-02-08

**Authors:** Milankumar Prajapati, Chelsea B. Quenneville, Jared Z. Zhang, Grace S. Chong, Lauren Chiu, Bangyi Ma, Lucas D. Ward, Ho-Chou Tu, Thomas B. Bartnikas

**Affiliations:** 1 Department of Pathology and Laboratory Medicine, Brown University, Providence, Rhode Island, USA; 2 Alnylam Pharmaceuticals, Cambridge, Massachusetts, USA

**Keywords:** manganese, metal, liver, toxicity, genetic disease, AAV, SLC30A10, T95I

## Abstract

The manganese (Mn) export protein SLC30A10 is essential for Mn excretion *via* the liver and intestines. Patients with SLC30A10 deficiency develop Mn excess, dystonia, liver disease, and polycythemia. Recent genome-wide association studies revealed a link between the SLC30A10 variant T95I and markers of liver disease. The *in vivo* relevance of this variant has yet to be investigated. Using *in vitro* and *in vivo* models, we explore the impact of the T95I variant on SLC30A10 function. While SLC30A10 I95 expressed at lower levels than T95 in transfected cell lines, both T95 and I95 variants protected cells similarly from Mn-induced toxicity. Adeno-associated virus 8-mediated expression of T95 or I95 SLC30A10 using the liver-specific thyroxine binding globulin promoter normalized liver Mn levels in mice with hepatocyte Slc30a10 deficiency. Furthermore, Adeno-associated virus–mediated expression of T95 or I95 SLC30A10 normalized red blood cell parameters and body weights and attenuated Mn levels and differential gene expression in livers and brains of mice with whole body Slc30a10 deficiency. While our *in vivo* data do not indicate that the T95I variant significantly compromises SLC30A10 function, it does reinforce the notion that the liver is a key site of SLC30A10 function. It also supports the idea that restoration of hepatic SLC30A10 expression is sufficient to attenuate phenotypes in SLC30A10 deficiency.

Manganese (Mn) is a dietary nutrient and essential metal (1, 2). It serves as a cofactor for many biological enzymes in key processes including growth and development, metabolic regulation, and immune function. Dietary Mn is absorbed by the small intestines, transported to the liver and other tissues, then excreted *via* hepatobiliary and intestinal excretion. Urinary excretion of Mn is minimal. The body maintains Mn at sufficient but nontoxic levels by specific homeostatic pathways. Over the past 10 years, studies in patients with rare, inherited diseases and animal models of these diseases have established three transport proteins, SLC39A14, SLC30A10, and SLC39A8, as key determinants of Mn homeostasis in the human body (3, 4, 5). All three proteins influence systemic Mn homeostasis by impacting Mn transport in hepatocytes and enterocytes. SLC39A14 imports Mn from blood into hepatocytes and enterocytes (6, 7, 8, 9, 10). SLC30A10 exports Mn from hepatocytes into bile and from enterocytes into the lumen of the gastrointestinal tract (11, 12). SLC39A8 imports Mn from bile into hepatocytes and may import Mn from the lumen of the gastrointestinal tract into enterocytes (13, 14, 15). Patients with SLC30A10 or SLC39A14 deficiency develop severe Mn excess and toxicity because of impaired Mn elimination from the body. While deficiency in SLC30A10 or SLC39A14 leads to neurological deficits, only SLC30A10 deficiency causes liver cirrhosis and polycythemia. Patients with SLC39A8 deficiency develop early-onset Mn deficiency and developmental defects secondary to excessive Mn loss from the body.

Recently, a rare missense T95I variant in SLC30A10 was linked to a variety of quantitative traits including increased serum alanine aminotransferase (ALT) and aspartate aminotransferase (AST) levels, risk of extrahepatic bile duct cancer, MRI-quantified hepatic inflammation, hemoglobin concentrations, and hematocrits (16, 17, 18). Notably, one study assessed plasma manganese levels in their cohort but did not find a significant association with T95I SLC30A10 among nine carriers tested (18). While a more recent report using cell culture models indicated that the T95I variant does not impact Mn transport by SLC30A10 (19), the impact of the T95I variant on SLC30A10 function *in vivo* has yet to be investigated. The goal of this study was to determine if the T95I variant impairs SLC30A10 function using both cell culture and mouse models of Slc30a10 deficiency. Furthermore, given that we used adeno-associated virus (AAVs) to overexpress SLC30A10 in livers of Slc30a10-deficient mice to study the impact of the T95I variant, our study also enabled us to explore the role of the hepatic SLC30A10 in Mn mobilization in the context of pre-existing Mn excess secondary to SLC30A10 deficiency.

## Results

### The I95 variant reduces SLC30A10 protein levels *via* proteasome-independent mechanisms but protects cells against Mn toxicity to a similar degree as wildtype T95

**Figure 1:**
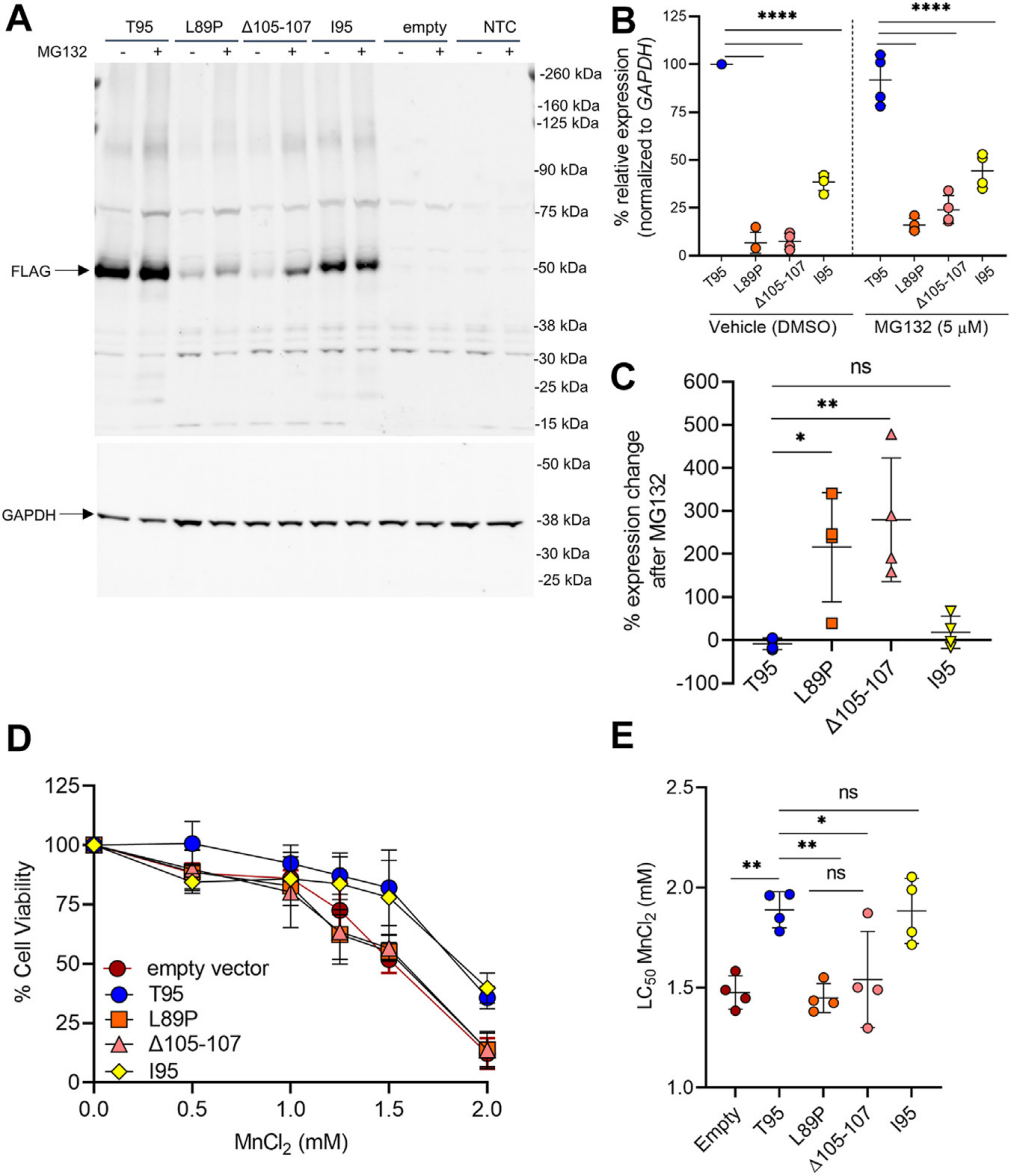
**The I95 variant reduces SLC30A10 protein levels *via* proteasome-independent mechanisms but protects cells against Mn toxicity to a similar degree as wildtype T95.***A–C,* HeLa cells were transiently transfected with plasmids overexpressing FLAG-tagged T95, L89P, Δ105 to 107, or I95 SLC30A10, empty vector, or no plasmid (NTC) for 24 to 30 h before treatment with 5 μM MG132 or vehicle (DMSO) for another 16 h. *A*, protein levels were assayed by Western blotting with anti-FLAG and anti-GAPDH antibodies. *B*, relative SLC30A10 protein levels were quantified and normalized to levels for the vehicle-treated T95 group. N = 4. *C*, percent change in SLC30A10 protein levels after MG132 treatment was calculated by normalizing to the protein levels of the respective vehicle-treated groups. *D* and *E*, HeLa cells were transiently transfected with FLAG-tagged SLC30A10 constructs as in (*A*) for 24 to 30 h and treated with 0 to 2.0 mM MnCl_2_ for 16 h. *D*, cell viability was evaluated with the MTT assay. *E,* LC_50_ values of cell viability assay, calculated by nonlinear regression. N = 4 for all groups except for 1.25 and 1.50 mM where N = 3. In (*B*, *C*, and *E*), one-way ANOVA with Tukey’s multiple comparisons test was used to determine statistical significance (∗*p* < 0.05; ∗∗*p* < 0.01; ∗∗∗∗*p* < 0.0001). Data are represented as mean ± standard deviation. Mn, manganese.

To explore the functional relevance of the SLC30A10 I95 variant, we first tested its stability and function in transiently transfected HeLa cells. We observed more than 50% reduction in I95 protein levels compared to T95 SLC30A10, while disease-associated variants (L89P, Δ105–107) were poorly expressed (Fig. 1, *A* and *B*). Treatment with the proteasomal inhibitor MG132 did not alter I95 protein levels (Fig. 1*C*). The I95 variant was as efficient as T95 SLC30A10 in protecting HeLa cells against Mn toxicity (LC_50_ 1.83 and 1.87 mM for I95 and T95, respectively), whereas cells expressing L89P or Δ105 to 107 showed lower LC_50_ values (LC_50_ 1.45 mM for both) (Fig. 1, *D* and *E*).

### AAV8-mediated hepatic SLC30A10 expression attenuates phenotypes in mice with hepatocyte Slc30a10 deficiency

**Figure 2:**
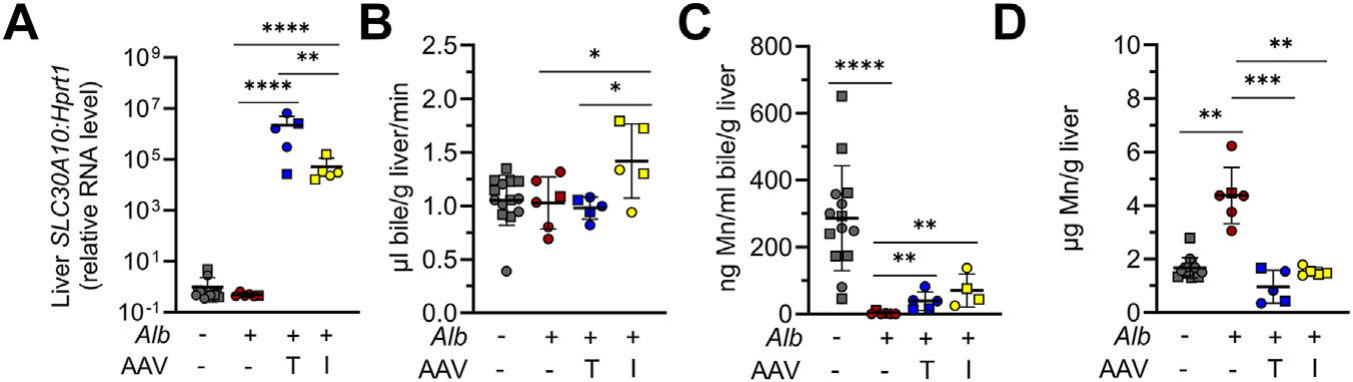
**AAV8-mediated SLC30A10 expression attenuates phenotypes in mice with hepatocyte Slc30a10 deficiency.***A–D,* two-month-old wildtype (*Slc30a10*^*lox/lox*^) and hepatocyte Slc30a10-deficient (*Slc30a10*^*lox/lox*^*Alb*) mice were injected retro-orbitally with saline or 2 × 10^11^ genome copies AAV8 carrying T95 (‘AAV T’) or I95 (‘AAV I’) human *SLC30A10* cDNA, then analyzed at 3 months of age for (*A*) liver *SLC30A10* RNA levels relative to *Hprt1* levels measured by QPCR, (*B*) bile flow rates, (*C*) bile Mn levels measured by GFAAS, and (*D*) liver Mn levels measured by ICP-OES. Data are represented as mean ± standard deviation with at least five samples and a roughly equal number of female and male mice per group. Data were first tested for normal distribution by Shapiro–Wilk test; if not normally distributed, data were log transformed. Groups were compared by two-way ANOVA with Tukey’s multiple comparisons test (∗*p* < 0.05; ∗∗*p* < 0.01; ∗∗∗*p* < 0.001; ∗∗∗∗*p* < 0.0001). Female and male data are indicated by *circles* and *squares,* respectively. AAV, adeno-associated virus; GFAAS, graphite furnace atomic absorption spectroscopy; ICP-OES, inductively coupled plasma–optical emission spectroscopy.

To investigate the *in vivo* impact of T95I on SLC30A10 function, we employed AAV8 vectors encoding T95 or I95 *SLC30A10* cDNA driven by the hepatocyte-specific thyroxine binding globulin (TBG) promoter. To confirm that the AAV8 vectors were functional, we studied their effect in mice with hepatocyte-specific Slc30a10 deficiency (*Slc30a10*^*lox/lox*^ *Alb*). These mice have minimal bile Mn levels and mildly elevated liver Mn levels (11). Mice were injected with saline or AAV carrying T95 or I95 *SLC30A10* cDNA at 1 month of age then analyzed 1 month later. Treatment with T95 and I95 AAV increased hepatic *SLC30A10* RNA levels in mutant mice, although the increase was more prominent for T95 (Fig. 2*A*). Treatment with I95 but not T95 AAV increased bile flow rates for reasons not clear (Fig. 2*B*). Treatment with either AAV increased bile Mn levels and normalized liver Mn levels to control levels (Fig. 2, *C* and *D*). These data suggested that the AAV vectors could be used to complement hepatic Slc30a10 deficiency in mice.

### AAV8-mediated hepatic SLC30A10 expression attenuates phenotypes in mice with whole body Slc30a10 deficiency

**Figure 3:**
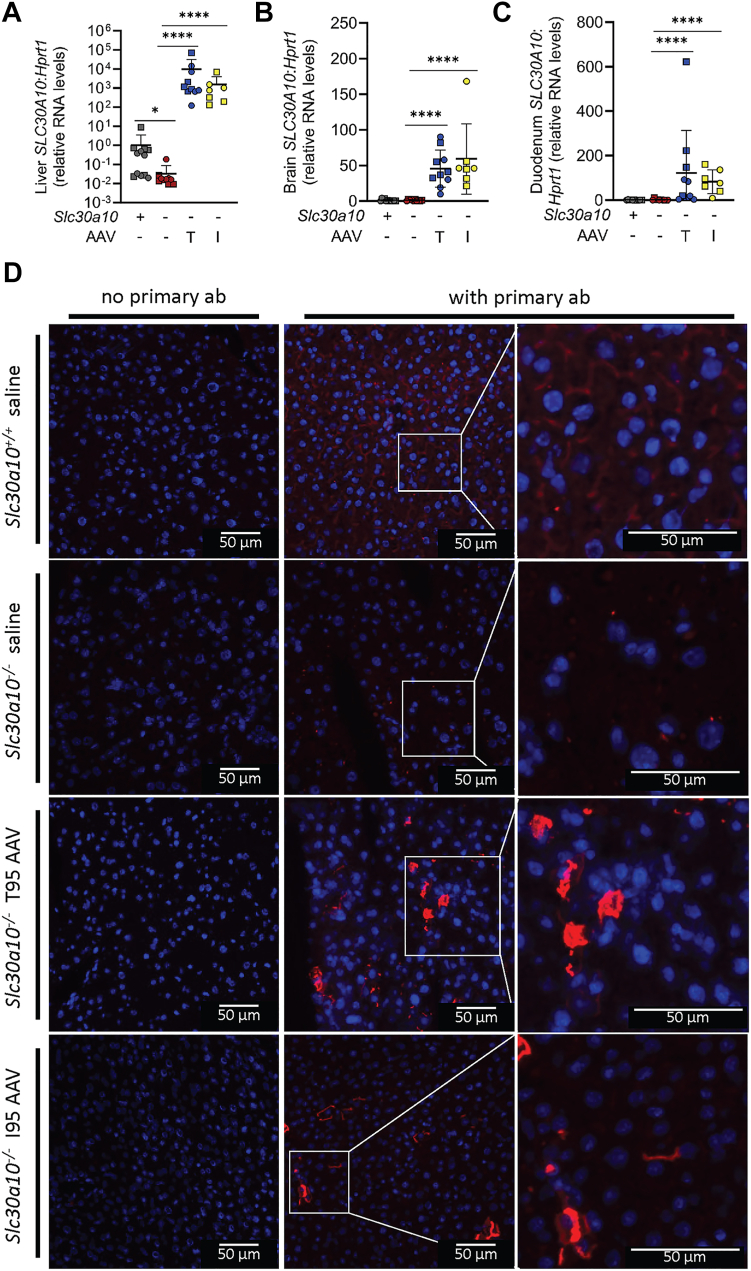
**AAV8 treatment increases SLC30A10 expression in *Slc30a10***^***−/−***^**mice.***A–D*, one-month-old wildtype (*Slc30a10*^*+/+*^, ‘*Slc30a10 +*’) and Slc30a10-deficient (*Slc30a10*^*−/−*^, ‘*Slc30a10 –‘*) mice were injected retro-orbitally with saline or 2 x 10^11^ genome copies AAV8 carrying T95 (‘AAV T’) or I95 (‘AAV I’) human *SLC30A10* cDNA, then analyzed at 2 months of age for liver (*A*), brain (*B*), and duodenum (*C*) *SLC30A10* RNA levels relative to *Hprt1* levels, as measured by QPCR; (*D*) SLC30A10 protein levels by immunofluorescence of frozen liver sections with DAPI staining for nuclei. (These and other images from this analysis can be found in Supplementary materials) Data represented and analyzed statistically as in Figure 2 (∗*p* < 0.05; ∗∗∗∗*p* < 0.0001). AAV, adeno-associated virus.

To further explore the functional relevance of the SLC30A10 T95I variant, we next studied mice with whole body Slc30a10 deficiency (*Slc30a10*^*−/−*^). Mice were injected with saline or AAV carrying T95 or I95 *SLC30A10* cDNA at 1 month of age then analyzed 1 month later. Treatment of mutant mice with T95 or I95 AAV increased *SLC30A10* RNA levels in liver, brain, and duodenum, although the increase was orders of magnitude greater in liver than brain and duodenum (Fig. 3, *A*–*C*). Immunofluorescence using anti-SLC30A10 antibodies demonstrated membrane staining in *Slc30a10*^*+/+*^ livers that was absent in *Slc30a10*^*−/−*^ livers and strong staining in a fraction of cells in livers of *Slc30a10*^*−/−*^ mice treated with either T95 or I95 AAV (Figs. 3*D* and S1–S5). In the latter group, immunofluorescent signal was much stronger than endogenous signal in *Slc30a10*^*+/+*^ livers and was not uniform across all cells—some cells exhibited punctate staining while others showed a membrane pattern.

**Figure 4:**
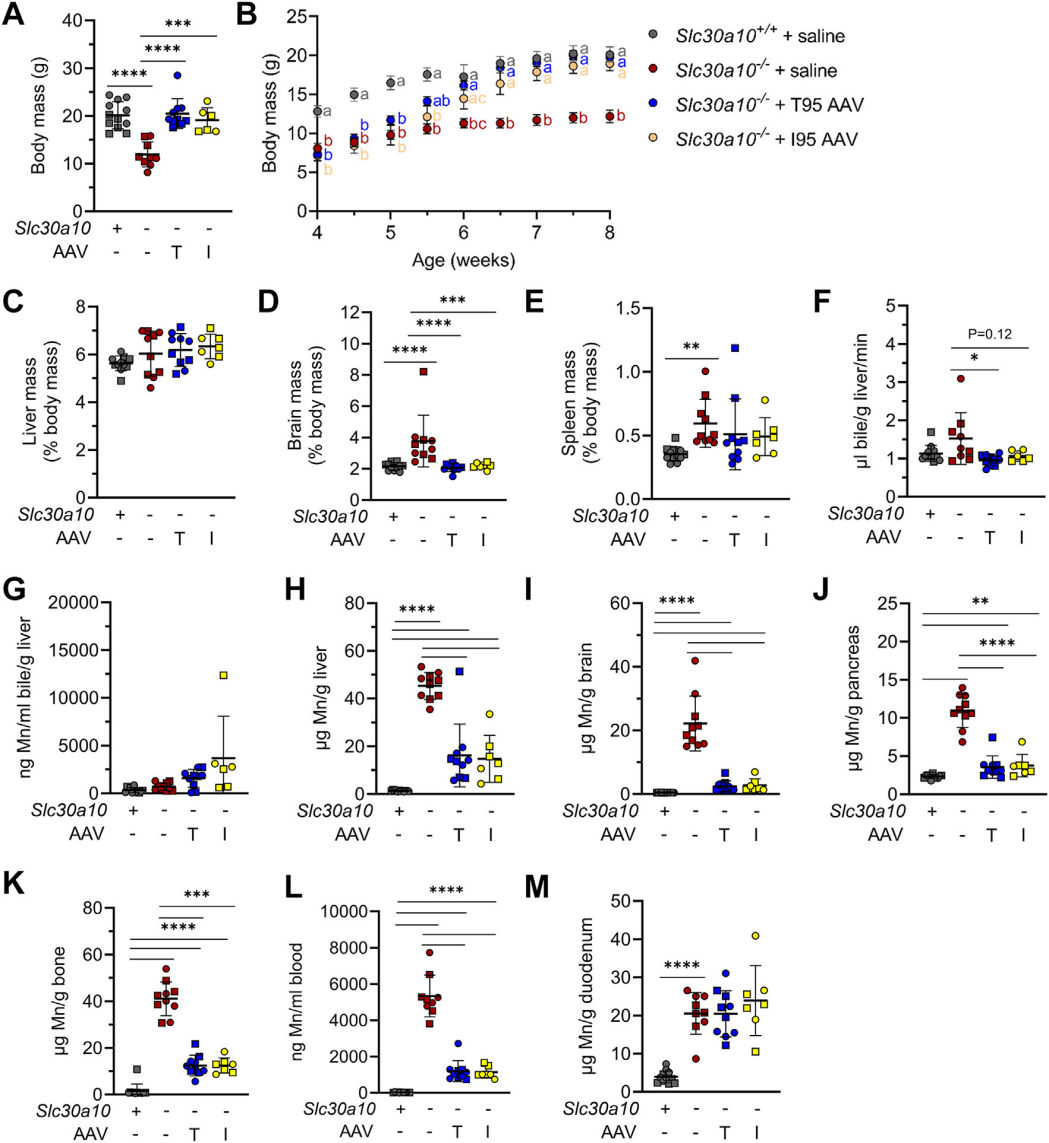
**AAV8-mediated SLC30A10 expression increases body mass and attenuates Mn excess in *Slc30a10***^***−/−***^**mice.***A–M*, mice from Figure 3 were analyzed for (*A*) body mass at time of harvest; (*B*) body mass over course of treatment; liver (*C*), brain (*D*), and spleen (*E*) mass relative to body mass; (*F*) bile flow rates; (*G*) bile Mn levels measured by GFAAS; liver (*H*), brain (*I*), pancreas (*J*), and bone (*K*) Mn levels measured by ICP-OES; (*L*) blood Mn levels measured by GFAAS; (*M*) duodenum Mn levels measured by ICP-OES. Data represented and analyzed statistically as in Figure 2. AAV, adeno-associated virus; ICP-OES, inductively coupled plasma–optical emission spectroscopy; Mn, manganese.

We have previously shown that *Slc30a10*^*−/−*^ mice have decreased body mass (11). Treatment of *Slc30a10*^*−/−*^ mice with either AAV normalized body mass (Fig. 4*A*). However, body masses did begin to increase half a week earlier in T95 than I95 AAV-treated mutant mice (Fig. 4*B*). Normalized body mass was not due to organomegaly—AAV treatment did not increase liver, brain, or spleen mass (Fig. 4, *C*–*E*).

*Slc30a10*^*−/−*^ mice also develop severe Mn excess although bile Mn levels are similar to wildtype mice (11). (We interpret these ‘normal’ bile Mn levels in *Slc30a10*^*−/−*^ mice as inappropriately low, given the severe Mn excess present in these mice.) T95 and I95 AAV treatment of mutant mice decreased bile flow rates to control levels, but the decrease only reached significance for T95 (Fig. 4*F*). AAV treatment also increased bile Mn levels, but the changes were not significant (Fig. 4*G*). Treatment with either AAV vector decreased Mn levels in liver, brain, pancreas, bone, and blood but had no impact on Mn levels in duodenum (Fig. 4, *H*–*M*).

**Figure 5:**
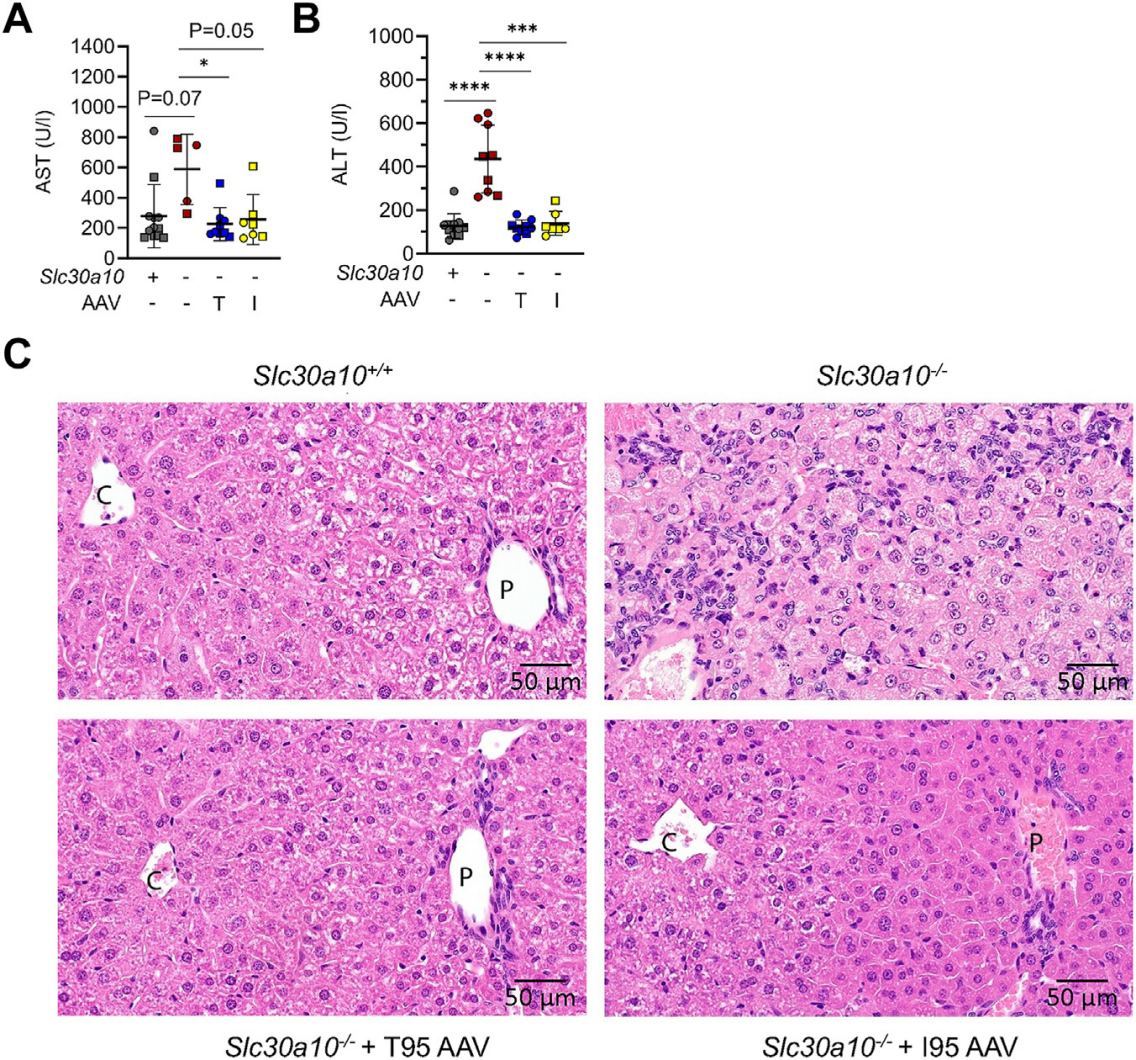
**AAV8-mediated SLC30A10 expression decreases serum AST and ALT levels and normalizes liver histology in *Slc30a10***^***−/−***^**mice.***A–C,* mice from Figure 3 were analyzed for (*A* and *B*) serum AST (*A*) and ALT (*B*) levels by AU480 Beckman Coulter chemistry analyzer; (*C*) liver histology by H&E staining of formalin-fixed tissues. Data in (*A* and *B*) represented and analyzed statistically as in Figure 2 (∗*p* < 0.05; ∗∗∗*p* < 0.001; ∗∗∗∗*p* < 0.0001). AAV, adeno-associated virus; ALT, alanine aminotransferase; AST, aspartate aminotransferase.

As mentioned above, the T95I variant has been linked to increased serum AST and ALT levels. Given this, we also analyzed AST and ALT levels in our mouse cohort. Treatment with either AAV vector normalized AST and ALT levels in mutant mice, although the increase in AST levels in AAV-untreated mice and the decrease in AST levels in I95-treated mice did not reach significance (Fig. 5, *A* and *B*). We also examined liver histology (Figs. 5*C* and S6–S13). Livers from saline-treated *Slc30a10*^*−/−*^ mice showed diffuse hepatocyte vacuolation, widespread hepatocyte single cell necrosis, and proliferation of oval and fibroblast-like nonparenchymal cells. Livers from T95 or I95 AAV-treated *Slc30a10*^*−/−*^ mice exhibited similar histology as *Slc30a10*^*+/+*^ mice.

**Figure 6:**
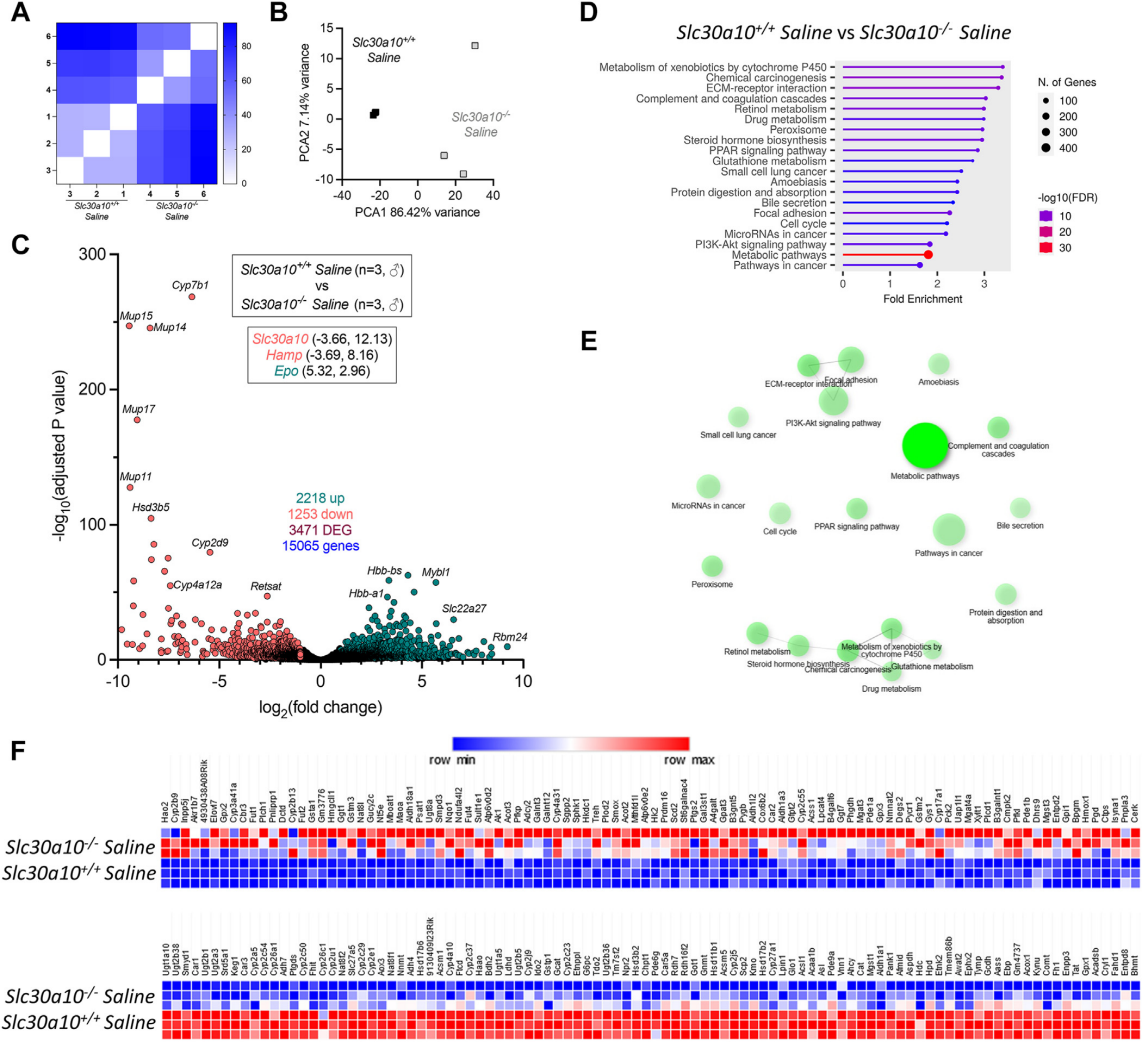
***Slc30a10***^***−/−***^**livers exhibit aberrant gene expression.** Bulk RNAseq was performed on livers from three male mice from each group shown in Figure 3. Shown here is comparison of *Slc30a10*^*+/+*^ and *Slc30a10*^*−/−*^ livers. *A*, similarity analysis. *B*, principal component analysis. *C,* volcano plot of genes differentially expressed between *Slc30a10*^*+/+*^ and *Slc30a10*^*−/−*^ livers. Differentially expressed genes (‘DEG’) (adjusted *p* value<0.05) are shown as *red* (log_2_(fold change) <1) or *green* (log_2_(fold change) >1) points with gene names shown adjacent as space permitted. Nondifferentially expressed genes are shown as *black points*. X-y coordinates of additional genes of interest shown in *smaller box*. Genes with log_2_(fold change) <0 are more abundantly expressed in *Slc30a10*^*+/+*^ mice; genes with log_2_(fold change) >0 are more abundantly expressed in *Slc30a10*^*−/−*^ livers. *D* and *E*, gene enrichment analysis. *F*, heat map of top 100 genes upregulated (*top*) and downregulated (*bottom*) in *Slc30a10*^*−/−*^ livers.

**Figure 7:**
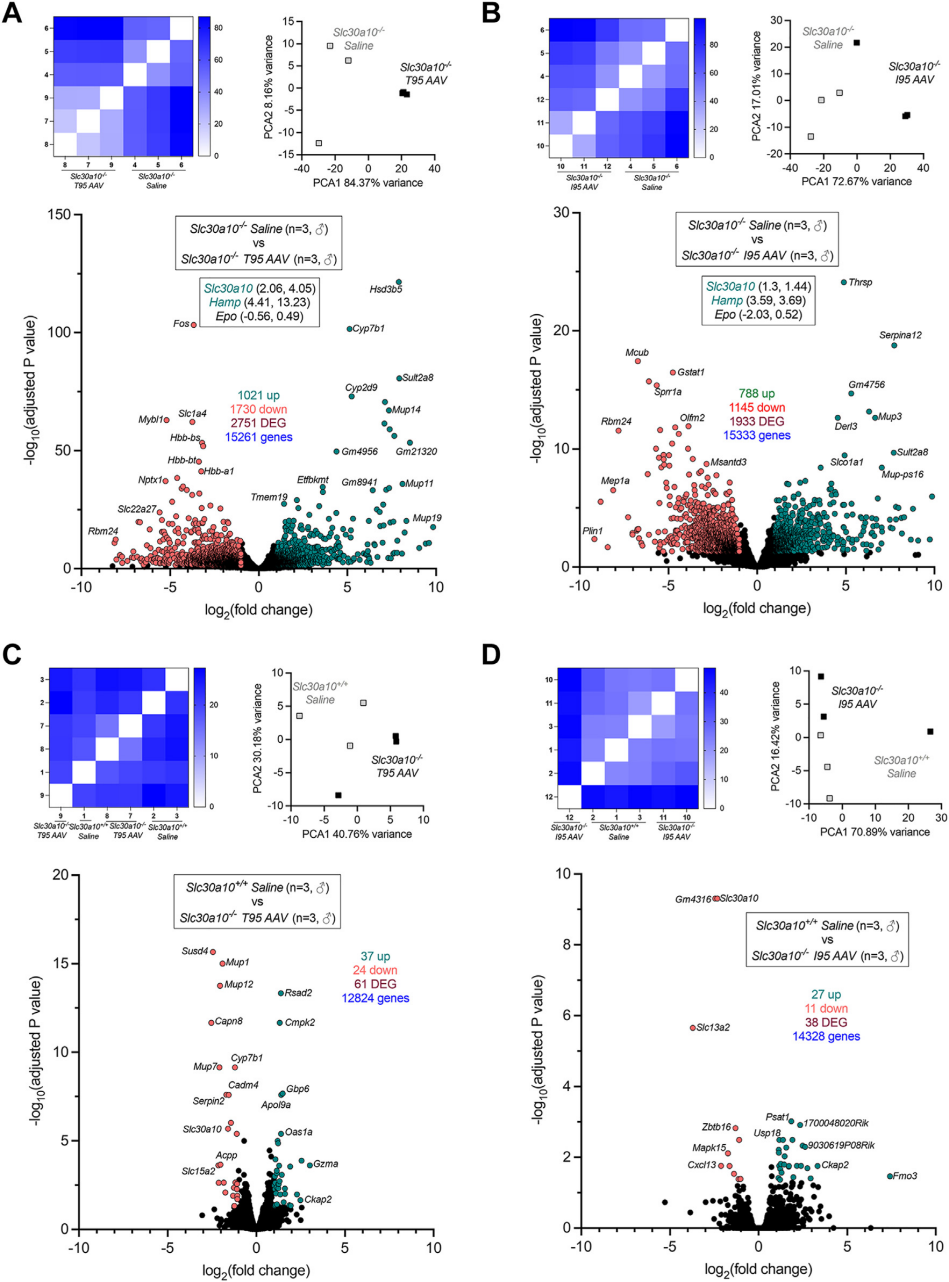
**Treatment with T95 or I95 AAVs attenuates aberrant gene expression in livers of *Slc30a10***^***−/−***^**mice.** Shown here are additional comparisons of RNAseq data from Figure 6.*A*, *Slc30a10*^*−/−*^*versus* T95 AAV-treated *Slc30a10*^*−/−*^; *B*, *Slc30a10*^*−/−*^*versus* I95 AAV-treated *Slc30a10*^*−/−*^; *C*, *Slc30a10*^*+/+*^*versus* T95 AAV-treated *Slc30a10*^*−/−*^; *D*, *Slc30a10*^*+/+*^*versus* I95 AAV-treated *Slc30a10*^*−/−*^. Results in each panel are depicted as in Figure 6, *A*–*C*. AAV, adeno-associated virus; DEG, differentially expressed gene.

To further assess the impact of AAV treatment on liver phenotypes, we performed bulk RNAseq on livers. Three thousand four hundred seventy one genes were differentially expressed in *Slc30a10*^*−/−*^ mice relative to *Slc30a10*^*+/+*^ mice (Fig. 6, *A*–*C*). These genes aligned with multiple pathways (Fig. 6, *D*–*F*). ‘Metabolic pathways’ was the most highly represented group, with genes upregulated and downregulated in this group. Treatment of *Slc30a10*^*−/−*^ mice with T95 and I95 AAV led to differential expression of 2751 and 1933 genes, respectively (Fig. 7, *A* and *B*). Comparison of T95 or I95 AAV samples to wildtype samples identified only 61 or 38 differentially expressed genes respectively, indicating that treatment with T95 and I95 AAV largely normalized gene expression in livers of mutant mice to a wildtype pattern (Fig. 7, *C* and *D*).

**Figure 8:**
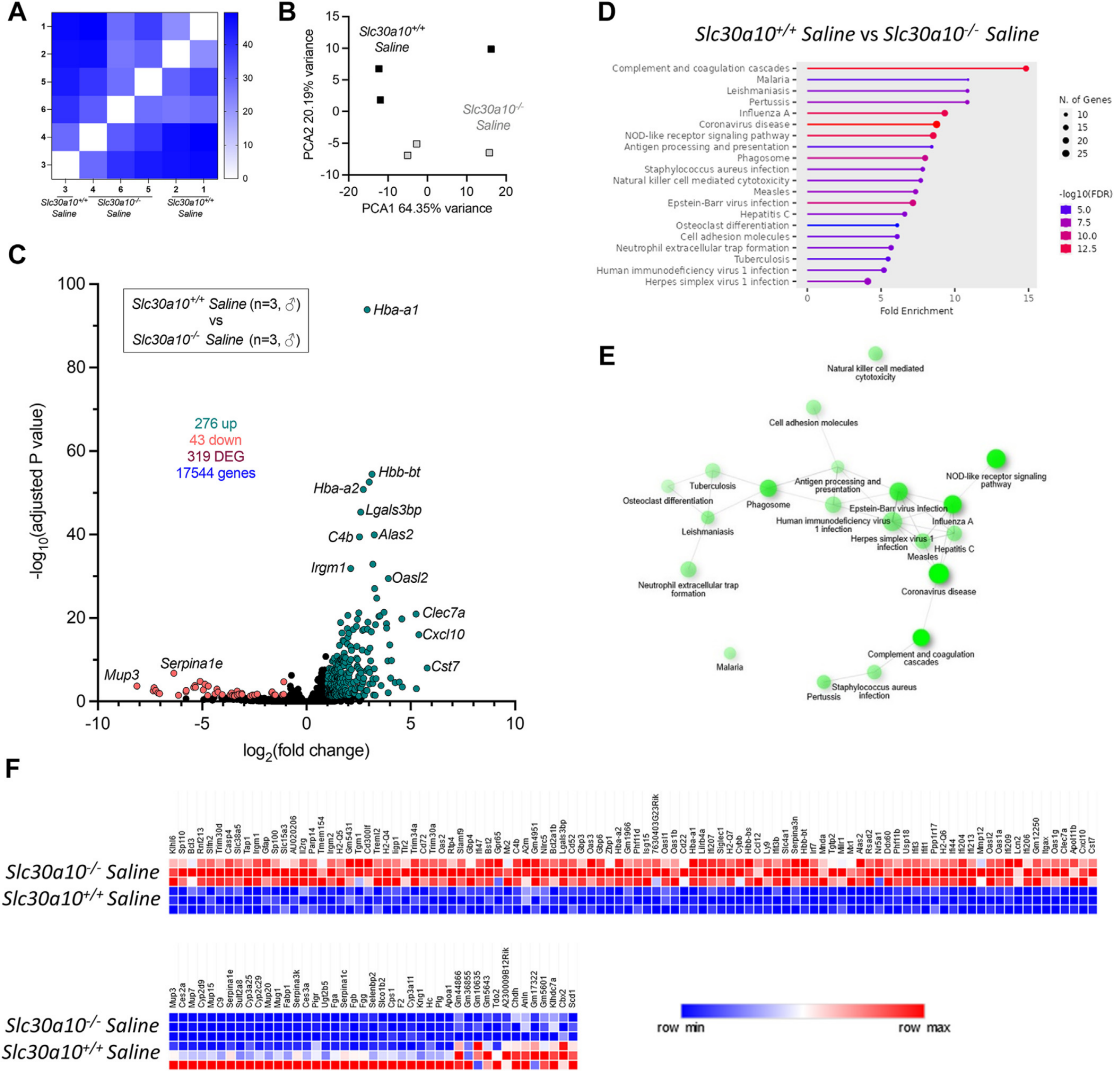
***Slc30a10***^***−/−***^**brains exhibit aberrant gene expression.** Bulk RNAseq was performed on brains from three male mice from each group shown in Figure 3. Shown here is comparison of *Slc30a10*^*+/+*^ and *Slc30a10*^*−/−*^ livers, depicted as in Figure 6. *A,* similarity analysis. *B*, principal component analysis. *C*, Volcano plot. *D* and *E,* gene enrichment analysis. *F*, heat map of top 100 genes upregulated (*top*) and downregulated (*bottom*) in *Slc30a10*^*−/−*^ brains. DEG, differentially expressed genes.

**Figure 9:**
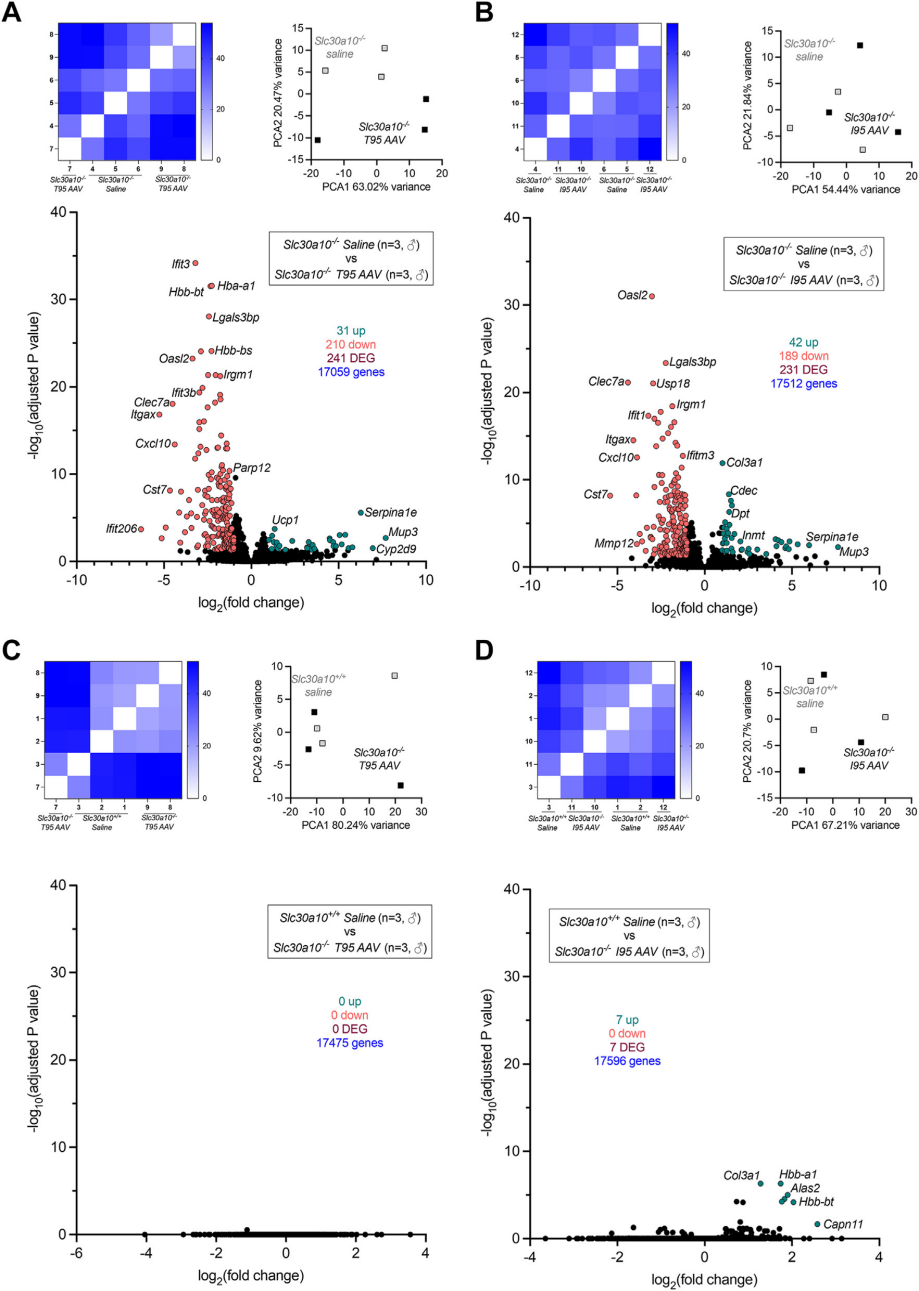
**Treatment with T95 or I95 AAVs attenuates aberrant gene expression in brains of *Slc30a10***^***−/−***^**mice.** Shown here are additional comparisons of RNAseq data from Figure 8.*A*, *Slc30a10*^*−/−*^*versus* T95 AAV-treated *Slc30a10*^*−/−*^; *B*, *Slc30a10*^*−/−*^*versus* I95 AAV-treated *Slc30a10*^*−/−*^; *C*, *Slc30a10*^*+/+*^*versus* T95 AAV-treated *Slc30a10*^*−/−*^; *D*, *Slc30a10*^*+/+*^*versus* I95 AAV-treated *Slc30a10*^*−/−*^. Results in each panel are depicted as in Figure 6, *A*–*C*. AAV, adeno-associated virus; DEG, differentially expressed gene.

We observed above that AAV treatment decreased brain Mn levels. To further assess brain phenotypes, we performed bulk RNAseq on whole brain samples. Three hundred nineteen genes were differentially expressed in *Slc30a10*^*−/−*^ mice relative to *Slc30a10*^*+/+*^ mice (Fig. 8, *A*–*C*). These genes aligned with multiple pathways, and most were upregulated in mutant mice (Fig. 8, *D*–*F*). Treatment of *Slc30a10*^*−/−*^ mice with T95 and I95 AAV led to differential expression of 241 and 231 genes, respectively (Fig. 9, *A* and *B*). Comparison of T95 or I95 AAV samples to wildtype samples identified 0 or seven differentially expressed genes respectively, indicating that treatment with T95 and I95 AAV largely normalized gene expression in brains of mutant mice to a wildtype pattern (Fig. 9, *C* and *D*).

**Figure 10:**
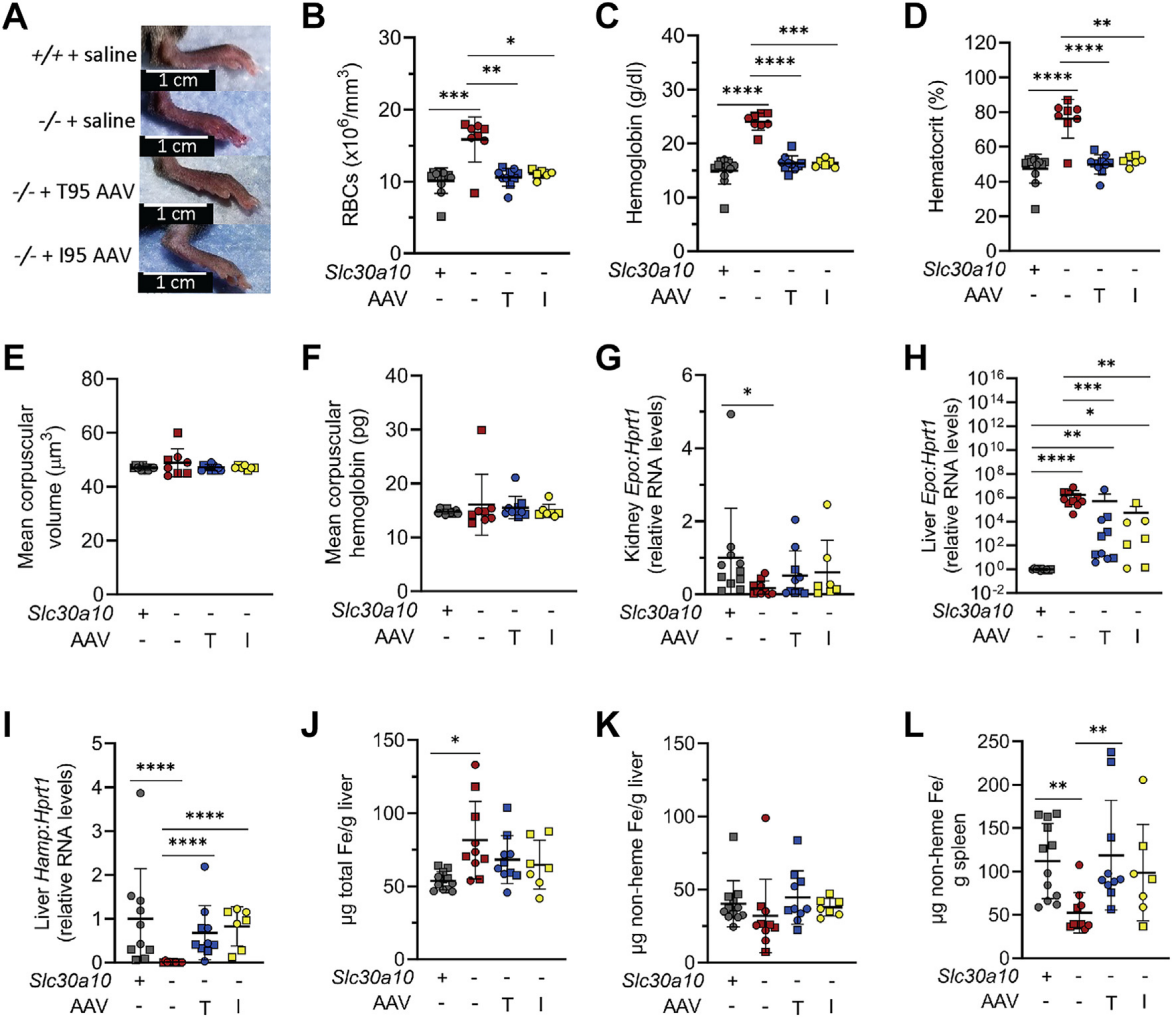
**AAV8-mediated SLC30A10 expression attenuates red blood cell and iron phenotypes in *Slc30a10***^***−/−***^**mice.***A–F,* mice from Figure 3 were analyzed for (*A*) images of paws; (*B*–*F*) red blood cell (RBC) counts (*B*), hemoglobin levels (*C*), hematocrits (*D*), mean corpuscular volumes (*E*), and mean corpuscular hemoglobin levels (*F*), as measured by complete blood counts; *G* and *H,* kidney (*G*) and liver (*H*) *Epo* RNA levels measured by QPCR; *I,* liver hepcidin RNA levels measured by QPCR; *J*, total iron (Fe) levels measured by ICP-OES; *K* and *L*, nonheme Fe levels in liver (*K*) and spleen (*L*), measured by bathophenanthroline-based assay. Data are represented as in Figure 2 (∗*p* < 0.05; ∗∗*p* < 0.01; ∗∗∗*p* < 0.001; ∗∗∗∗*p* < 0.0001). AAV, adeno-associated virus.

*Slc30a10*^*−/−*^ mice also develop increased red blood cell (RBC) counts (11). Paws in all AAV-treated *Slc30a10*^*−/−*^ mice were less reddish in color (Fig. 10*A*). Treatment with either AAV normalized RBC counts, hemoglobin levels, and hematocrits (Fig. 10, *B*–*D*). Mean corpuscular volumes and mean corpuscular hemoglobin levels were not increased in *Slc30a10*^*−/−*^ mice or affected by AAV treatment (Fig. 10, *E* and *F*). Patients with SLC30A10 deficiency also develop erythropoietin (EPO) excess. EPO is hormone that stimulates erythropoiesis and is produced by the kidneys under physiologic conditions but can be synthesized by the liver under pathologic conditions (20). *Epo* RNA levels were decreased in kidneys but increased in livers in untreated mice (Fig. 10, *G* and *H*). (Serum Epo levels were not measured, as inadequate amounts of serum remained after AST and ALT assessments.) Treatment of mutant mice with either AAV increased kidney *Epo* RNA levels insignificantly and decreased liver *Epo* RNA levels significantly (Fig. 10, *G* and *H*). In addition to stimulating erythropoiesis, EPO also indirectly suppresses hepatic expression of hepcidin, a hormone that inhibits dietary iron absorption and macrophage iron release (21). Liver hepcidin RNA levels were decreased in untreated mutant mice and increased in AAV-treated mice (Fig. 10*I*). To examine the impact of aberrant hepcidin levels on tissue iron levels in *Slc30a10*^*−/−*^ mice, we next measured liver and spleen iron levels. Total iron levels were increased in untreated mutant mice relative to wildtype mice, while nonheme iron levels did not differ between any group (Fig. 10, *J* and *K*). This is consistent with excess iron residing in red blood cells in polycythemic mice. Spleen nonheme iron levels were decreased in untreated mutant mice relative to wildtype mice (Fig. 10*L*). This observation is consistent with hepcidin deficiency, given that the spleen is rich in red cell-scavenging macrophages, and hepcidin inhibits iron export from these cell types.

## Discussion

While deleterious mutations in *SLC30A10* cause severe disease, studies have linked SLC30A10 polymorphisms with markers of disease and other parameters of health. For example, epidemiological studies have reported an association between SLC30A10 polymorphisms, Mn levels, and neurological function in otherwise healthy individuals (22, 23). As discussed above, the T95I SLC30A10 variant has been linked to multiple phenotypes in several studies of human populations. This variant resides on the third of six transmembrane domains in the protein, which also harbors loss-of-function variants L89P and Δ105 to 107. To investigate the potential impact of this variant on SLC30A10 function, we performed *in vitro* and *in vivo* studies. Our *in vitro* data demonstrated that the I95 variant is expressed at lower levels than the T95 form and that this reduction is independent of the proteasome. However, both T95 and I95 SLC30A10 protected cells against Mn toxicity similarly, suggesting, at least in a transfected cell model, a negligible impact of the T95I variant on SLC30A10 function. Our *in vivo* analyses employed AAV8 vectors and TBG promoters to drive SLC30A10 expression in mice with hepatocyte and whole body Slc30a10 deficiency. While *SLC30A10* I95 RNA levels were lower than T95 RNA levels, the majority of phenotypes assayed in mutant mice were impacted similarly by expression of T95 or I95 SLC30A10. These phenotypes included body mass; Mn levels; serum AST and ALT levels; liver histology; differential gene expression in liver and brain; RBC parameters; *Epo* and hepcidin RNA levels; and liver and spleen Fe levels. Overall, our results are in agreement with the cell culture–based report that the T95I variant has negligible impact on Mn transport by SLC30A10 (19). In this work, Gurol *et al.* found no impact of the T95I variant on Mn levels, protection against Mn toxicity, Mn transport, or protein stability in HeLa and HepG2 cell lines (19).

Beyond our analysis of the *in vivo* impact of the T95I variant on SLC30A10 function, our study has implications for our understanding of Mn homeostasis. Hepatobiliary excretion is well-established as the central route of elimination of Mn from the body. We previously demonstrated in mouse models that intestinal Slc30a10 mediates Mn export from the blood to the lumen of the gastrointestinal tract (11), but our current study suggests that Mn excretion by enterocyte Slc30a10 is not a main route of excretion and most likely only represents an alternative route of excretion when hepatobiliary excretion is compromised. Our study has also implications for the treatment of SLC30A10 deficiency. While AAV treatment did increase *SLC30A10* RNA levels in extrahepatic organs, our data suggest that increasing liver SLC30A10 expression can reduce systemic Mn excess in *Slc30a10*^*−/−*^ mice. This implies that correcting SLC30A10 deficiency in the liver is sufficient to attenuate severity of disease. As such, AAV-driven expression of SLC30A10 in the liver may represent a potential treatment for patients with SLC30A10 deficiency.

While hepatic expression of wildtype SLC30A10 had a prominent impact on disease characteristics in mutant mice, not all phenotypes corrected to wildtype levels. For example, we observed normalization of red blood cell parameters even though Mn levels in treated *Slc30a10*^*−/−*^ mice did not correct to wildtype levels. One explanation for this observation is that there is a threshold for Mn levels below which certain disease phenotypes no longer manifest. We also noted that liver *Epo* RNA levels in AAV-treated *Slc30a10*^*−/−*^ mice did not correct to *Slc30a10*^*+/+*^ levels to the same extent that RBC counts, hematocrits, or hemoglobin levels did. This could reflect the possibility that there is a threshold of liver *Epo* RNA levels below which polycythemia no longer occurs in mutant mice, but this threshold is greater than liver *Epo* RNA levels observed in wildtype mice. Another potential issue here is that liver *Epo* RNA levels may not reflect serum Epo levels in our mouse model. We did prioritize measuring serum AST and ALT levels in our mice given that the T95I variant has been associated with AST and ALT levels, but this resulted in insufficient volumes of serum for Epo measurements.

While we did take an *in vivo* approach to studying the impact of the T95I variant on SLC30A10 function, there are limitations to our study. First, AAV-mediated transduction can lead to protein overexpression. SLC30A10 expression levels were much higher in AAV-treated *Slc30a10*^*−/−*^ mice than in untreated *Slc30a10*^*+/+*^ mice. Immunofluorescence of livers from AAV-treated mice indicated that not all hepatocytes were transduced and that cellular immunofluorescent patterns were not similar across all transduced cells across a tissue section or even between mice in the same treatment group. It is possible that the I95 variant has impaired function relative to T95, yet overexpression in our model rendered us unable to detect differences in function between T95 and I95 SLC30A10. Knocking in the T95I variant into the endogenous *Slc30a10* gene would circumvent this issue. Second, increased *SLC30A10* RNA levels were noted in the intestines of AAV-treated *Slc30a10*^*−/−*^ mice, which is not surprising given that extrahepatic expression with AAV8-TBG vectors has been reported previously (24). Given that enterocyte Slc30a10 exports Mn into the gastrointestinal tract, we cannot definitively conclude that hepatic SLC30A10 expression was solely responsible for attenuation of phenotypes in AAV-treated *Slc30a10*^*−/−*^ mice. Third, our study focuses largely on the impact of SLC30A10 expression on Mn levels. Notably, a recent report did not find an association between T95I and plasma Mn levels in a general population (18). Furthermore, the T95I variant may have Mn-independent effects on SLC30A10, as recently proposed by Gurol *et al*. in their cell culture-based study of SLC30A10 T95I (19). This group put forth several possibilities explaining their finding that the T95I variant has no discernible impact on SLC30A10 function. First, they noted that the T95I variant may impact Mn transport in cell types other than hepatocytes such as cholangiocytes. While AAV8 has been reported to transduce cholangiocytes (25), AAV8 avidly infects hepatocytes and as such our approach would not be able to differentiate impacts of the T95I variant in hepatocytes *versus* other cell types. As proposed by Gurol *et al.* and by us above, the appropriate next step would be to introduce the T95I variant into the endogenous mouse *Slc30a10* gene. However, this approach may not be successful if any adverse effects of the T95I variant depend on interactions with residues that diverge between the mouse and human protein sequence or with unconserved noncoding elements. Another possibility raised by Gurol *et al.* is that SLC30A10 has Mn-independent roles. In this case, the T95I variant would adversely impact these Mn-independent roles while not compromising Mn transport. This possibility is supported by a recent report documenting homozygous SLC30A10 W275G variants in patients with neurological symptoms but without polycythemia or hypermanganesemia (26)—in these patients, neurological deficits are noted in the absence of blood Mn excess, suggesting that SLC30A10 mutations can have adverse effects without prominently perturbing Mn levels. While the notion of Mn-independent functions of SLC30A10 is an intriguing one, such roles have yet to be identified.

Finally, we would like to point out that while most of our phenotypic characterization focused on Mn levels in AAV-treated mice, we also carried out unbiased transcriptomic analyses of liver and brain. Our goal with these analyses was to determine if expression of T95 and I95 SLC30A10 variants had equal impact on end-organ function, but it also enabled us to determine the impact of Slc30a10 deficiency on gene expression in these organs (without AAV treatment). Comparison of liver transcriptomes between *Slc30a10*^*+/+*^ and *Slc30a10*^*−/−*^ mice identified almost 3500 differential expressed genes which aligned to multiple pathways. ‘Metabolic pathways’ was the group that aligned with the greatest number of differentially expressed genes. The relevance of this finding certainly deserves future investigation, given that liver failure is one of the known consequences of SLC30A10 deficiency in patients. This RNA-seq finding could reflect hepatotoxicity of excess Mn, a role for Slc30a10 in regulating the multitude of metabolic pathways carried out by the liver, an adaptive response by the liver to detrimental effects of Slc30a10 deficiency such as Mn excess or a combination of these and other factors. Transcriptomic changes in the liver could also reflect the loss of Mn-independent roles of Slc30a10. One approach to identifying these impacts would be to raise Slc30a10-deficient mice on Mn-deficient diets and repeating the bulk RNA-seq analysis, as well as performing single-cell RNA-seq to identify cell type-specific, Mn-independent roles for Slc30a10.

Another pathway differentially represented between *Slc30a10*^*+/+*^ and *Slc30a10*^*−/−*^ livers was ‘bile secretion’. We did assess bile flow rates during our bile collection surgeries and noted that bile flow rates were increased in mutant mice then decreased when mutant mice were treated with T95 or I95 AAV, although most changes did not reach statistical significance (Fig. 4*F*). This finding does suggest that Slc30a10 deficiency impacts bile secretion. Another link between bile and Slc30a10 has been previously reported—in 2020, Ahmad *et al.* published that the bile acid lithocholic acid regulates intestinal Slc30a10 expression *via* vitamin D receptor signaling. Could bile acids regulate Slc30a10 expression in the liver as well? Furthermore, could Slc30a10 regulate bile secretion? This could be explored by profiling bile acid content in bile from *Slc30a10*^*+/+*^ and *Slc30a10*^*−/−*^ mice as well as examining the regulation of Slc30a10 expression in hepatocytes, cholangiocytes, and other cell types by bile acids.

## Experimental procedures

### Cell culture

HeLa cells (ATCC) were grown in Eagle’s Minimum Essential Medium (ATCC) containing 10% fetal bovine serum (Gibco) at 37 °C and 5% CO_2_. All plasmid transfections were performed using Lipofectamine 2000 (Invitrogen) and Opti-MEM (Gibco) according to manufacturer’s specifications. FLAG-tagged SLC30A10 plasmid constructs designed with a linker sequence in pCMV6-AN-3DDK (Blue Heron Biotech) included T95 (wildtype), P89 (p.Leu89Pro), Δ105-107 (p.Ala105_Pro107del), and I95 (p.Thr95Ile) *SLC30A10* and used an empty vector for one of the negative controls. The other negative control was a nontemplate control which contained only Lipofectamine 2000 and Opti-MEM.

### Immunoblot analysis

HeLa cells were grown on 6-well plates overnight and transfected with plasmids overexpressing SLC30A10 wildtype and variants for 24 to 30 h. Cells were then treated with 5 μM MG132 or vehicle (DMSO) (EMD Millipore Corp.) for another 16 h before the cells were harvested for Western blot analysis. Cell pellets were lysed with RIPA buffer (Thermo Scientific) containing protease inhibitor (Thermo Scientific) for 1 h on ice. Total protein was measured with the BCA assay (Thermo Scientific), and lysates were mixed with NuPAGE LDS (Invitrogen) containing DTT (Life Technologies) before running through a 4 to 12% Bis-Tris gel (Invitrogen) with the Chameleon Duo Pre-stained Protein Ladder (Licor). Proteins were transferred to a PVDF membrane (Invitrogen) with the iBlot2 system. Western blots were blocked for 1 h at room temperature in a buffer containing Odyssey Blocking Buffer (Licor) and PBS. After blocking, the Western blots were incubated overnight at 4 °C in a buffer containing Odyssey Blocking Buffer and PBST (G-Biosciences) with their corresponding primary antibody, rabbit anti-human FLAG antibody (dilution 1:500; Sigma-Aldrich), or mouse anti-human GAPDH antibody (dilution 1:500; Santa Cruz Biotechnology, Inc.) for detecting FLAG-tagged SLC30A10 or GAPDH, respectively. The western blots were rinsed three times in PBST and then incubated for 1 h at room temperature in their corresponding IRDye 800CW secondary antibody (dilution 1:5000; Licor), donkey anti-rabbit, or donkey anti-mouse for detecting FLAG-tagged SLC30A10 or GAPDH, respectively. The secondary antibody buffer contained Odyssey Blocking Buffer, PBST, and SDS (Invitrogen). Blots were then rinsed three times in PBST and imaged with the Bio-Rad ChemiDoc MP system. Protein levels were quantified with the Image Lab Touch Software which detected the protein band intensities.

### Cell viability assay

HeLa cells were plated on 6-well plates overnight and transfected with plasmids expressing SLC30A10 wildtype and variants for approximately 30 h. The cells were then harvested and replated at 10,000 cells/well in a 96-well plate for 24 h before cells were treated with 0 to 2 mM MnCl_2_ (Sigma-Aldrich) for 16 h. Cell viability was then assayed with CellTiter 96 Non-Radioactive Cell Proliferation (MTT) Assay (Promega) following the manufacturer’s specifications. Triton X-100-treated negative control wells were used to control for background absorbance caused by cell debris.

### Generation of mice, AAV8-TBG treatment, and sample collection

Animal work was approved by the Institutional Animal Care and Use Committee at Brown University. Heterozygous mice (*Slc30a10*^*+/−*^, C57BL/6N background) were bred together to generate wildtype (*Slc30a10*^*+/+*^) and mutant (*Slc30a10*^*−/−*^) mice. To generate mice with hepatocyte-specific Slc30a10 deficiency (*Slc30a10*^*lox/lox*^ *Alb+*), *Slc30a10*^*lox/lox*^ mice were bred to C57BL/6NJ mice expressing an albumin promoter-driven Cre recombinase (Jackson Laboratories) as described earlier (11). Mice were housed in ventilated cage racks, maintained on a 12-h light/dark cycle with controlled temperature and humidity, and provided standard chow (LabDiet 5010; 120 ppm Mn) and water *ad libitum*. Littermates were randomly assigned to experimental groups containing N of 5 to 10 mice. Adeno-associated viruses (AAV8 serotype) overexpressing either SLC30A10 T95 or I95 under control of the TBG promoter were provided by Alnylam Inc as ready-to-use vials. rAAV was produced by transient HEK 293 cell transfection and CsCl sedimentation by the University of Massachusetts Medical School Viral Vector Core, as previously described (27). Vector preparations were determined by ddPCR, and purity was assessed by SDS-PAGE and silver staining. AAV8-TBG viral particles were delivered *via* retro-orbital injection in single dose of 2 x 10^11^ genome copies per mouse under anesthesia. One month after AAV8-TBG injections, mice underwent bile, blood, and tissue collection as previously described (11), with bile collected over 60 min. Blood was collected *via* inferior vena cava puncture into EDTA-coated tubes (BD) for CBC analysis, then into serum collection tubes (BD). Mice were euthanized by cervical dislocation followed by tissue collection for biochemical analysis. Tissue samples were weighed and flash frozen into liquid nitrogen and stored at −80 °C until analysis. RBC parameters were measured by Scil VetAbcPlus CBC analyzer.

### QPCR

Total RNA was isolated using TRIzol reagent (Invitrogen). 1 μg DNase-treated RNA was used for cDNA synthesis using High-Capacity cDNA Reverse Transcription kit (Applied Biosystems). QPCR was carried out on ViiA7 Real-Time PCR System (Applied Biosystems) using PowerUp SYBR Green Master Mix (Applied Biosystems). RNA concentrations were determined using standard curve quantification approach followed by normalization using reference genes (*Hprt1*). Primer sequences used for gene-specific QPCR were tested for specificity using BLAST search and validated empirically by agarose gel electrophoresis and DNA sequencing of amplicons. The following primer pairs were used: *Hprt1* (5′-GCCCCAAAATGGTTAAGGTT, 5′-TGGCAACATCAACAGGACTC, spanning exons 6–9); *SLC30A10* (5′-TGTCATCTGCCTTCCCGCTTATC, 5′-CTAATTCCAGGCACAGCAGAGAG-, spanning exons 3–4); *Epo* (5′ACTCTCCTTGCTACTGATTCCT, 5′ATCGTGACATTTTCTGCCTCC, spanning exons 2–3); *Hamp* (5′TGTCTCCTGCTTCTCCTCCT, 5′CTCTGTAGTCTGTCTCATCTGTTG, spanning exons 1–2). *SLC30A10* QPCR without reverse transcriptase were used to detect any genomic DNA contamination or viral DNA from AAV treatments.

### Metal analysis

Total levels of tissue Mn and iron were measured as described earlier (11). Briefly, 50 to 200 mg tissues were digested in trace metal grade nitric acid at 65 °C for 2 h, followed by 25-fold dilution in MilliQ water. Diluted samples were analyzed by inductively coupled plasma–optical emission spectroscopy (Thermo Fisher Scientific, iCAP 7400 DUO). A series of standards were analyzed and used to extrapolate metal levels in the samples. Three mock digests were included in each run to control for metal contamination. A quality control standard (IV-28, Inorganic Ventures) was run every 10th sample to correct for variations in sensitivity throughout the run. Blood and bile Mn levels were measured by graphite furnace atomic absorption spectroscopy (PerkinElmer AAnalyst 600). Briefly, blood Mn levels were measured by digestion with 1:2 ratio of blood:trace metal grade nitric acid (Fisher) at 65 °C for 2 hours followed by 25-fold dilution with MilliQ water (Millipore Sigma). Bile Mn levels were measured by two rounds of digestion with equal volume of Optima nitric acid (Fisher) at 65 °C until dried, followed by equal volume of Optima hydrogen peroxide (Fisher) at 65 °C until dried. The dried samples were resuspended in 2% nitric acid at 50-fold final dilution followed by analysis using graphite furnace atomic absorption spectroscopy.

For measurement of tissue nonheme iron levels, 10 to 200 mg tissue was digested in 1 ml 3 N hydrochloric acid (Fisher)/10% trichloroacetic acid (Millipore Sigma) at 65 °C for 2 days, with 30 min vortexing each day, followed by centrifugation. Iron levels were measured by mixing 10 μl supernatants with 200 μl chromagen [five volumes MilliQ water; five volumes saturated sodium acetate (Fisher); one volume chromagen stock, consisting of 0.1% bathophenanthroline sulfonate (Millipore Sigma) and 1% thioglycolic acid (Millipore Sigma)] in a 96-well plate. Iron standards (Fisher) were included. After a 10-minute incubation, absorbances were measured at 535 nm. Mock digests without samples were included for this and all other metal analyses.

### AST and ALT measurements

Serum samples were tested at Investigation Toxicology Lab in Alnylam. ALT and AST reagents were purchased from Beckman Coulter. Two levels of quality control were purchased from Bio-Rad. Samples were run on AU480 Chemistry Analyzer (Beckton Coulter) after passage of two levels of quality control.

### RNA sequencing

RNA extraction, library preparation, sequencing, and analysis was conducted at Azenta Life Sciences (South Plainfield) as follows. Total RNA was extracted from fresh frozen tissue samples using Qiagen Rneasy Plus Universal mini kit following manufacturer’s instructions (Qiagen). RNA samples were quantified using Qubit 2.0 Fluorometer (Life Technologies), and RNA integrity was checked using Agilent TapeStation 4200 (Agilent Technologies). RNA sequencing libraries were prepared using the NEBNext Ultra II RNA Library Prep for Illumina using manufacturer’s instructions (NEB). Briefly, mRNAs were initially enriched with Oligod(T) beads. Enriched mRNAs were fragmented for 15 min at 94 °C. First strand and second strand cDNA were subsequently synthesized. cDNA fragments were end repaired and adenylated at 3′ends, and universal adapters were ligated to cDNA fragments, followed by index addition and library enrichment by PCR with limited cycles. The sequencing libraries were validated on the Agilent TapeStation (Agilent Technologies) and quantified by using Qubit 2.0 Fluorometer (Invitrogen) as well as by quantitative PCR (KAPA Biosystems). The sequencing libraries were clustered on a flowcell. After clustering, the flowcell was loaded on the Illumina instrument (4000 or equivalent) according to manufacturer’s instructions. The samples were sequenced using a 2 x 150 bp Paired End configuration. Image analysis and base calling were conducted by the Control software. Raw sequence data (.bcl files) generated the sequencer were converted into fastq files and de-multiplexed using Illumina’s bcl2fastq 2.17 software. One mismatch was allowed for index sequence identification. After investigating the quality of the raw data, sequence reads were trimmed to remove possible adapter sequences and nucleotides with poor quality. The trimmed reads were mapped to the reference genome available on ENSEMBL using the STAR aligner v.2.5.2 b. The STAR aligner is a splice aligner that detects splice junctions and incorporates them to help align the entire read sequences. BAM files were generated as a result of this step. (BAM files were uploaded on the NCBI Sequence Read Archive under accession PRJNA924303.) Unique gene hit counts were calculated by using feature Counts from the Subread package v.1.5.2. Only unique reads that fell within exon regions were counted. After extraction of gene hit counts, the gene hit counts table was used for downstream differential expression analysis. Using DESeq2, a comparison of gene expression between the groups of samples was performed. The Wald test was used to generate *p*-values and Log2 fold changes. Genes with adjusted *p*-values < 0.05 and absolute log2 fold changes > 1 were called as differentially expressed genes for each comparison. A PCA analysis was performed using the “plotPCA” function within the DESeq2 R package. The top 500 genes, selected by highest row variance, were used to generate PCA plots. Venn diagrams were generated using bioinformatics.psb.ugent.be/webtools/Venn/. Gene ontology was performed using ShinyGO at http://bioinformatics.sdstate.edu/go/ (28). Heatmaps on rlog-transformed gene counts were generated using Morpheus at https://software.broadinstitute.org/morpheus/.

### Immunofluorescence

Immunofluorescence was performed as described earlier (11). Briefly, 8-μm frozen sections were fixed in ice-cold methanol for 10 min followed by washing with PBS and blocking (blocking buffer: 10% goat serum, 1% BSA in PBS, 0.3% Triton X-100) for 1 hour at room temperature. Sections were incubated overnight with or without SLC30A10 antibody (Abcam AB229954, lot#GR3216193–24) in blocking buffer at 4 °C. Slides were washed in PBS followed by incubation with a secondary antibody (goat anti–rabbit IgG, Alexa Fluor 594 (ThermoFisher) in blocking buffer (without Triton X-100) for 2 hours at room temperature. Slides were washed in PBS and mounted using VECTASHIELD Vibrance Antifade Mounting Medium with DAPI (Vector Laboratories). All incubations were carried out in a humidified chamber with minimized light exposure to avoid photobleaching. Slides were imaged using Nikon Ti2-E Fluorescence Microscope.

### Statistical analysis

Statistics for all studies except RNAseq were performed using GraphPad Prism 9 (v 9.3.1), and the data were analyzed as previously reported (29). Data were first tested for normal distribution by Shapiro–Wilk test; if not normally distributed, data were log transformed. Groups were compared by 1-, 2-, or 3-way ANOVA with Tukey’s multiple comparisons test. *p* <0.05 was considered significant. LC_50_ values were calculated by nonlinear regression (curve fit) analysis. Data are shown as means ± standard deviation in the figures.

## Data availability

Further information and requests for resources and raw data should be directed to thomas_bartnikas@brown.edu. BAM files were deposited at NCBI SRA under BioProject accession number PRJNA1003945.

## Supporting information

This article contains Supporting information.

## Conflict of interest

The authors declare that they have no conflicts of interest with the contents of this article.

## Supporting information

Supplementary information **Figures S1-S5. Liver Slc30a10 protein levels in AAV-treated mice.** Frozen liver sections from mice from Fig. 3 were analyzed by immunofluorescence with an anti-SLC30A10 antibody (“primary”) and DAPI staining. Immunofluorescence was also performed without antibody (“no primary”) for reference. AAV, adeno-associated virus. **Figures S6-S13. Liver histology in AAV-treated mice.** Fixed liver sections from mice from Figure 3 were analyzed with H&E staining. AAV, adeno-associated virus.
