# Supplementary material for: AAV-mediated hepatic expression of SLC30A10 and the Thr95Ile variant attenuates manganese excess and other phenotypes in Slc30a10-deficient mice
Source: J Biol Chem. 2024 Feb 8;300(3):105732. doi: 10.1016/j.jbc.2024.105732 (PMC10933546; doi:10.1016/j.jbc.2024.105732)
Supplement: Supplementary information — Figures S1-S5. Liver Slc30a10 protein levels in AAV-treated mice. Frozen liver sections from mice from Fig. 3 were analyzed by immunofluorescence with an anti-SLC30A10 antibody (“primary”) and DAPI staining. Immunofluorescence was also performed without antibody (“no primary”) for reference. AAV, adeno-associated virus. Figures S6-S13. Liver histology in AAV-treated mice. Fixed liver sections from mice from Figure 3 were analyzed with H&E staining. AAV, adeno-associated virus. [file mmc1.pdf]

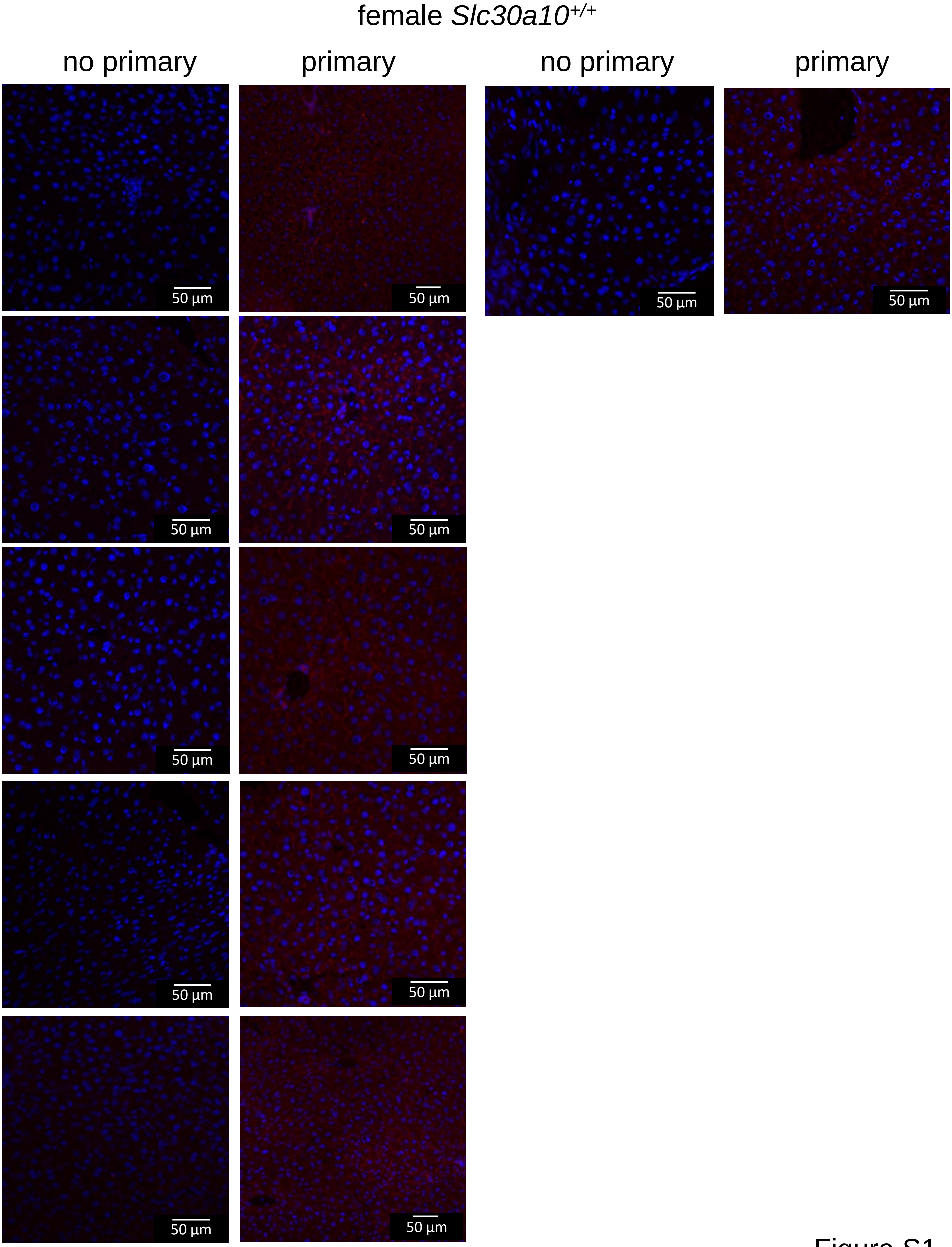
female Slc30a10+/+
no primary
primary
no primary
primary
50 µm

Figure S1

male $Slc30a10^{+/+}$

no primary | primary | no primary | primary

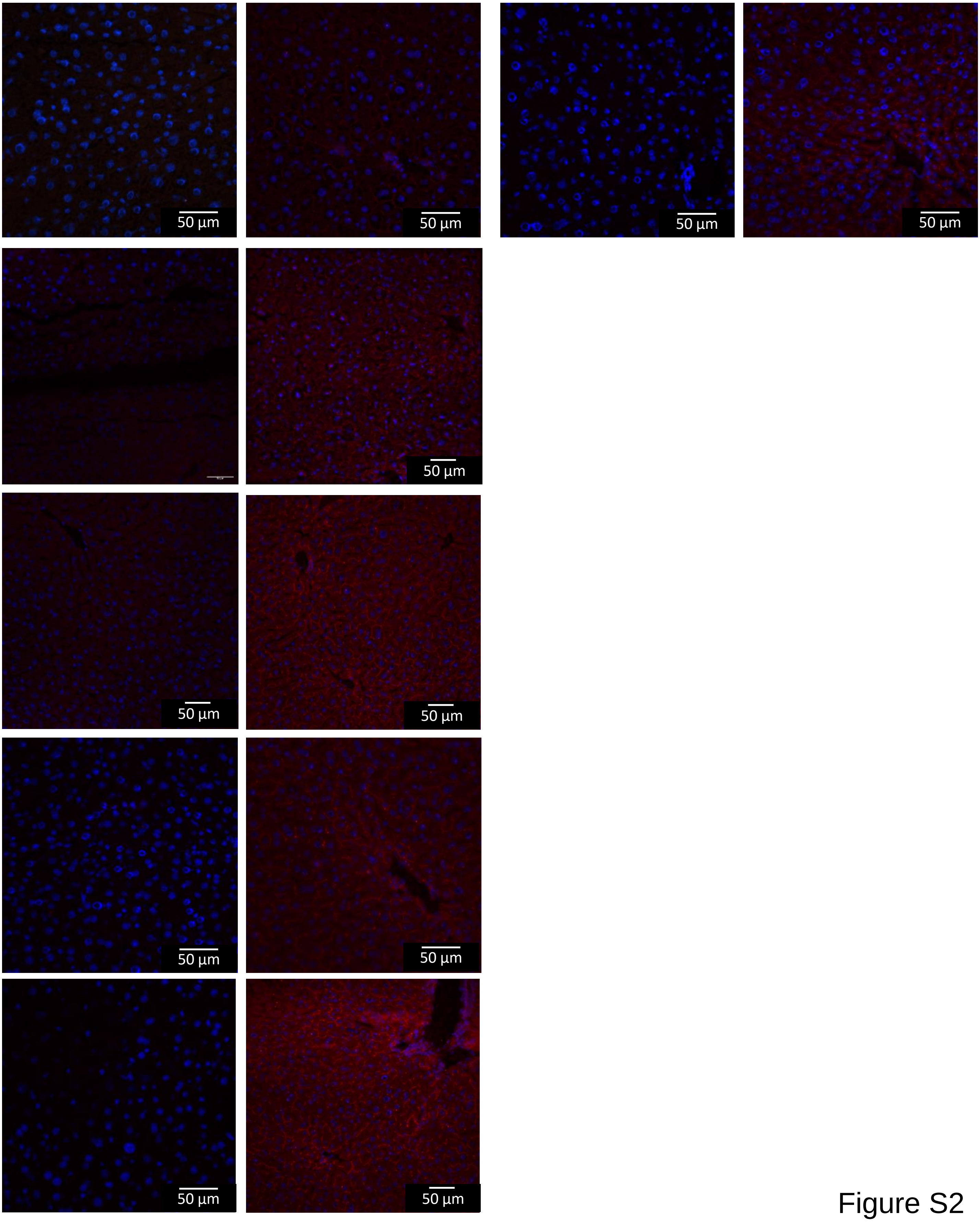

Figure S2

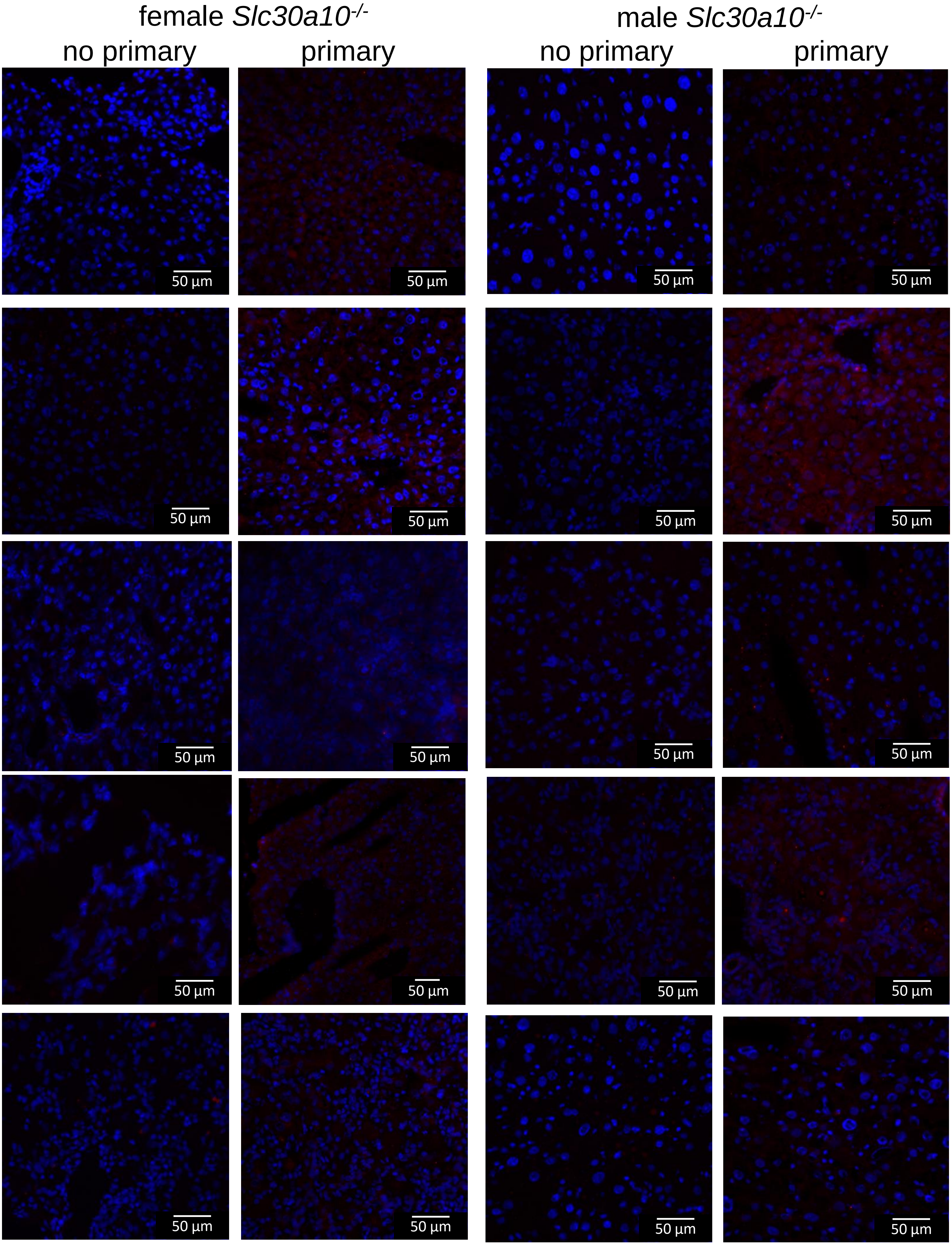
female Slc30a10-/-
male Slc30a10-/-
no primary
primary
no primary
primary
50 µm

Figure S3

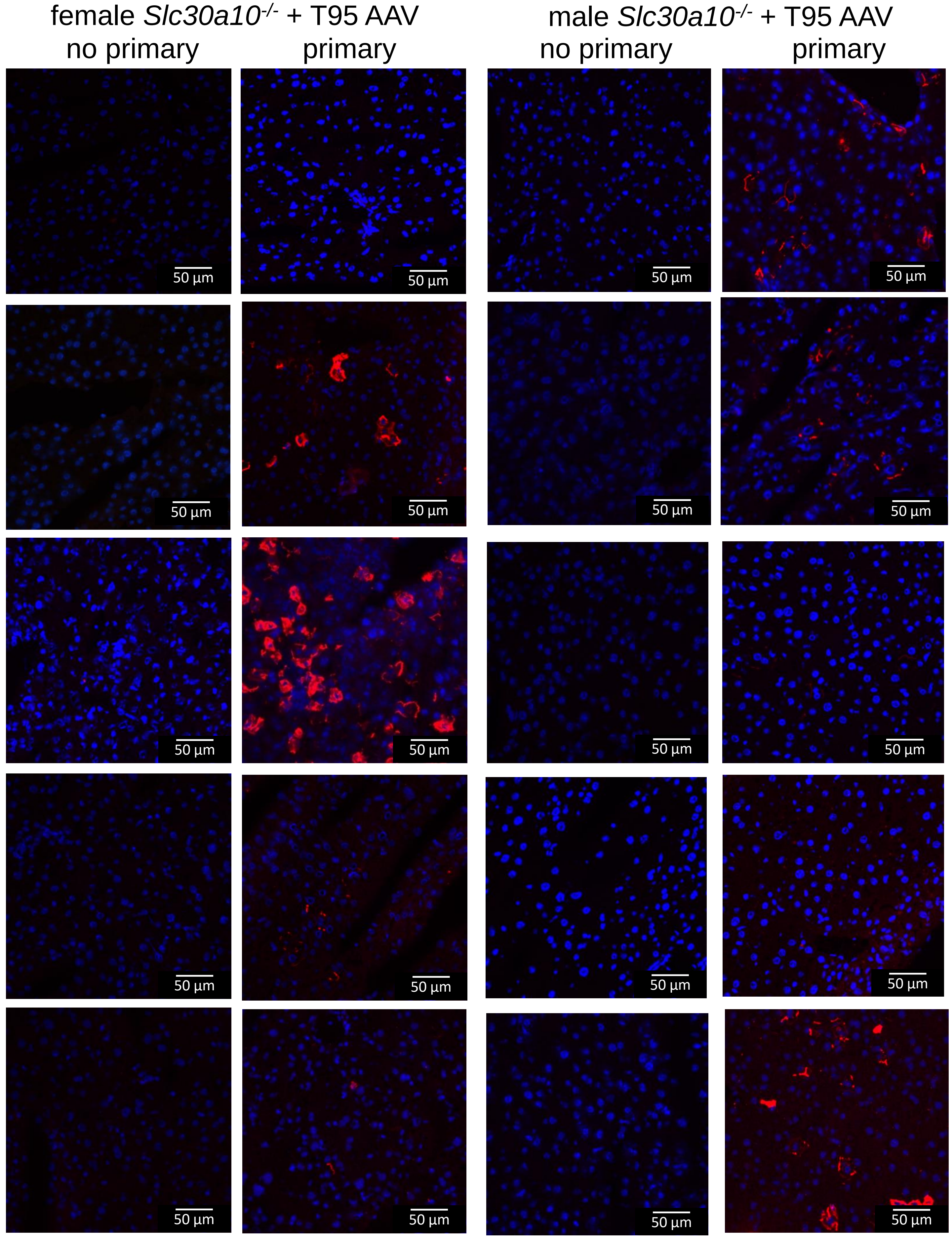
female $Slc30a10^{-/-}$ + T95 AAV
no primary
primary
male $Slc30a10^{-/-}$ + T95 AAV
no primary
primary
50 µm

Figure S4

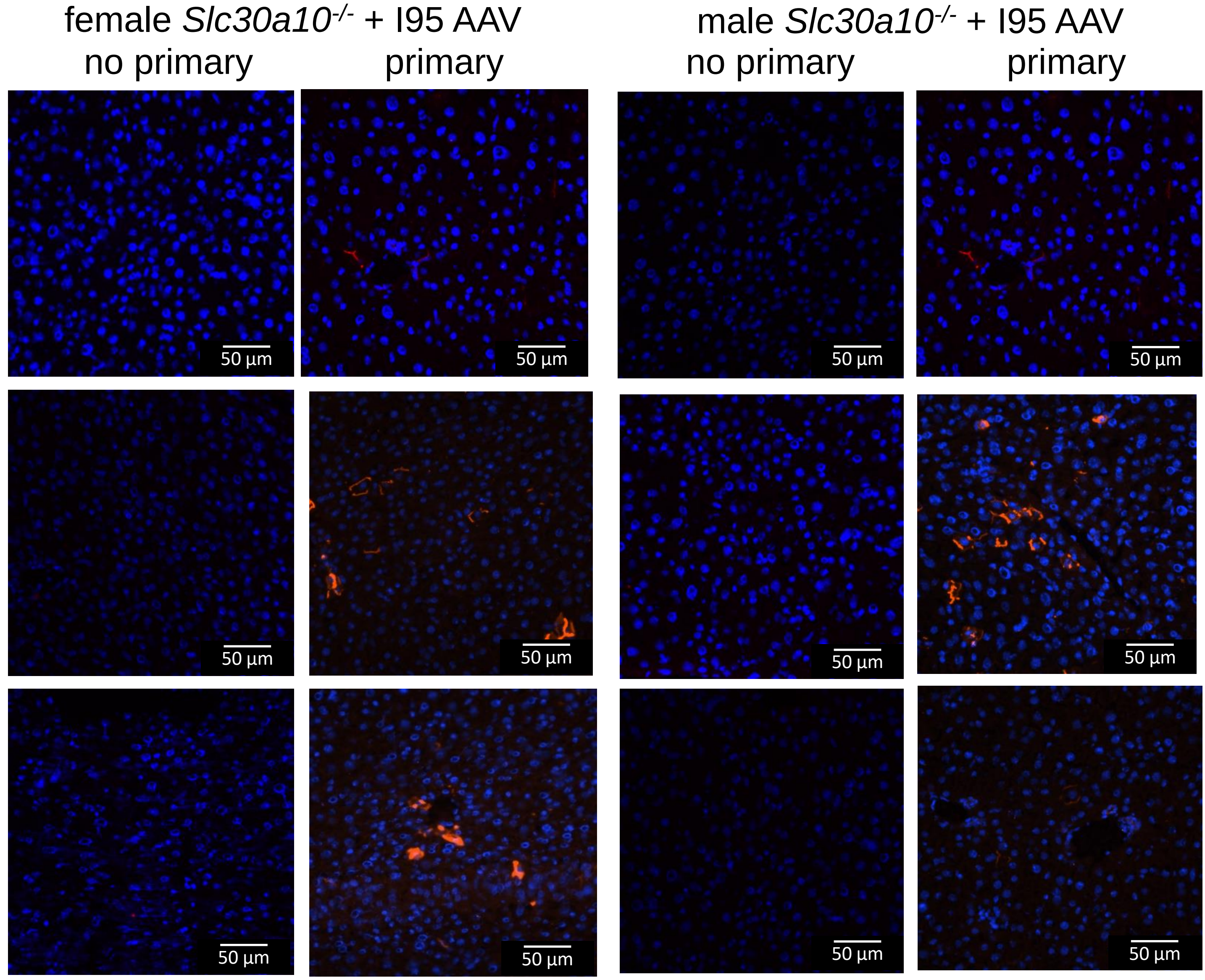
female Slc30a10-/- + I95 AAV
no primary
primary
male Slc30a10-/- + I95 AAV
no primary
primary
50 µm

Figure S5

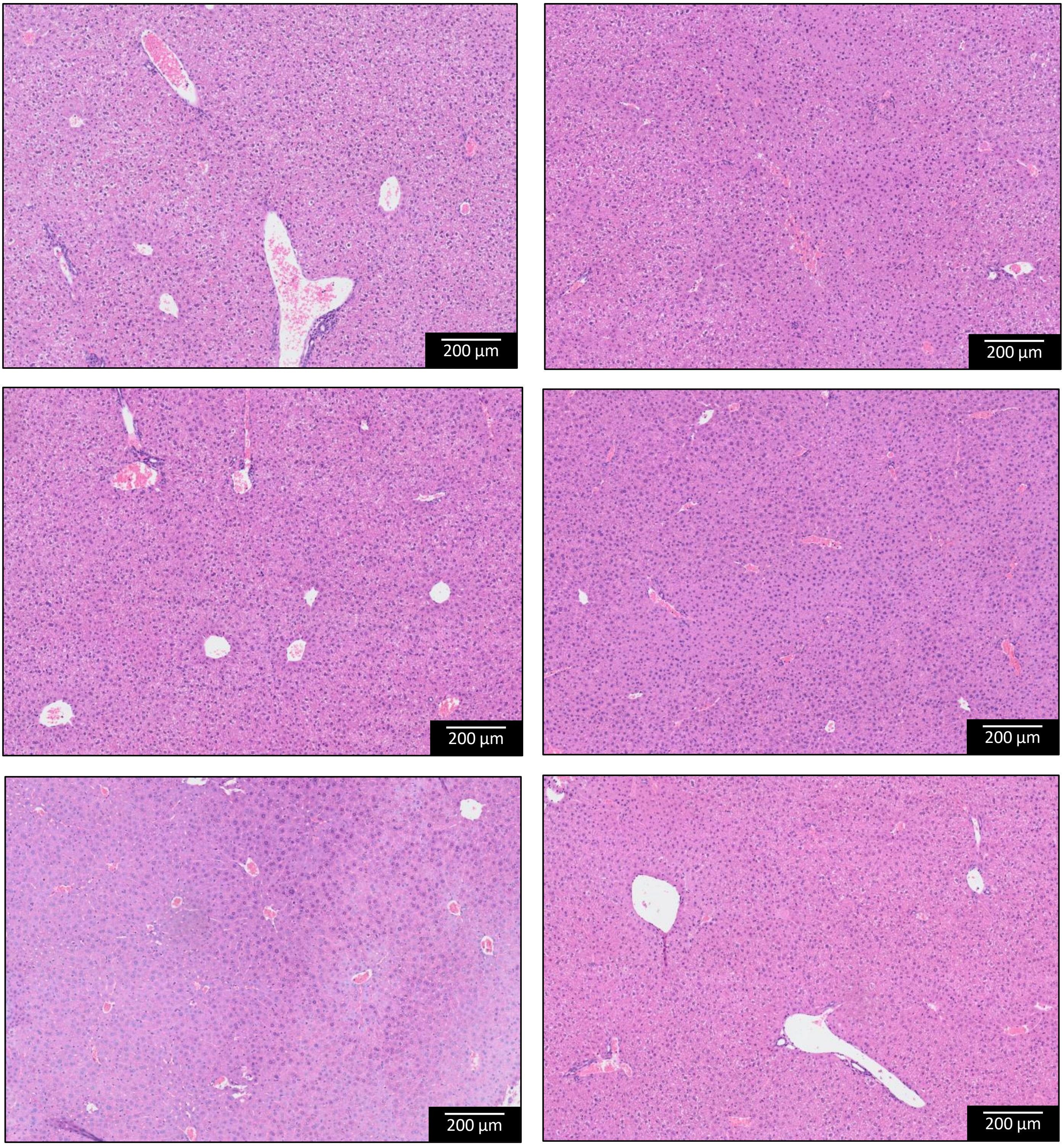

female *Slc30a10*$^{+/+}$

Figure S6

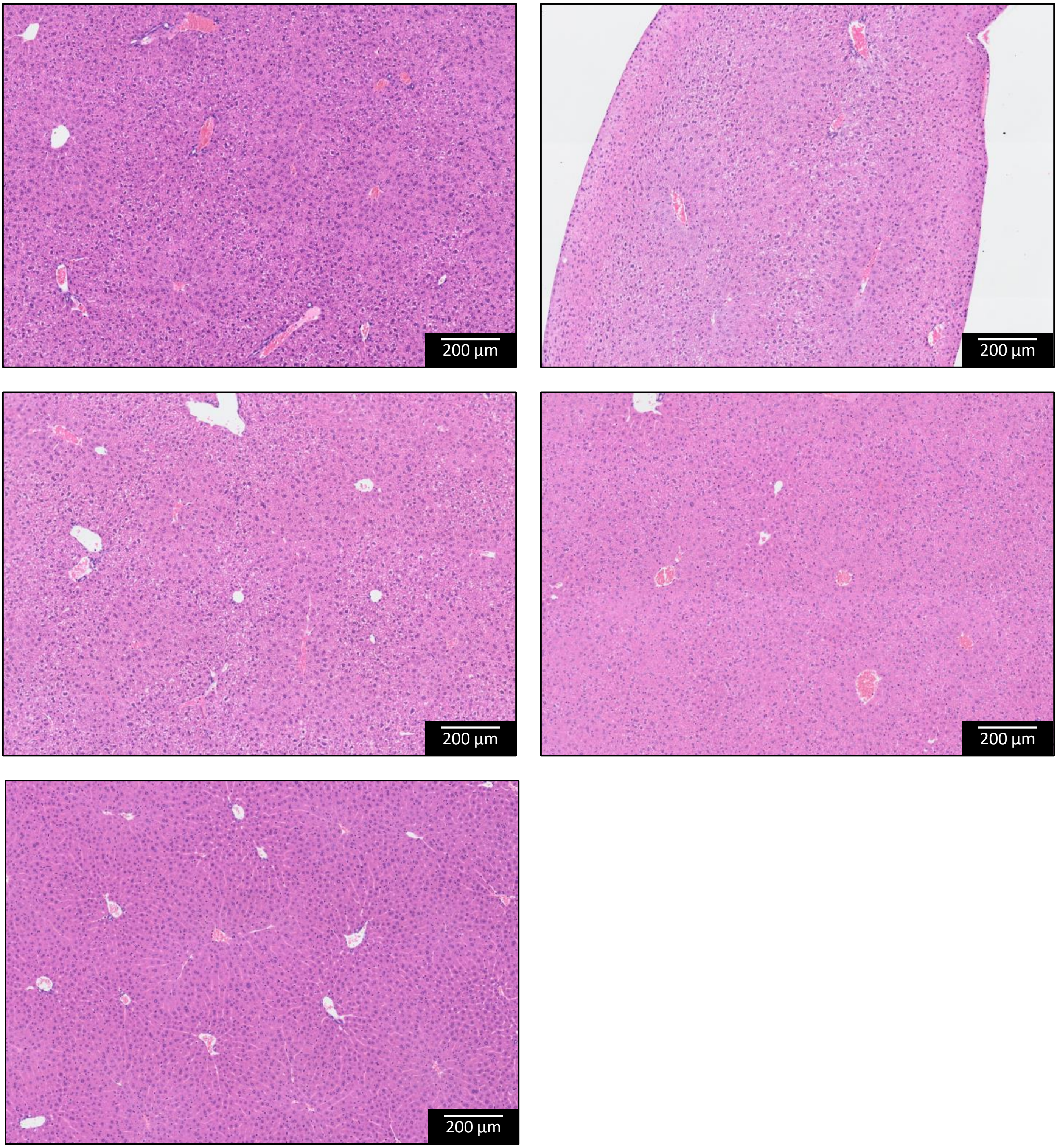

male *Slc30a10*$^{+/+}$ Figure S7

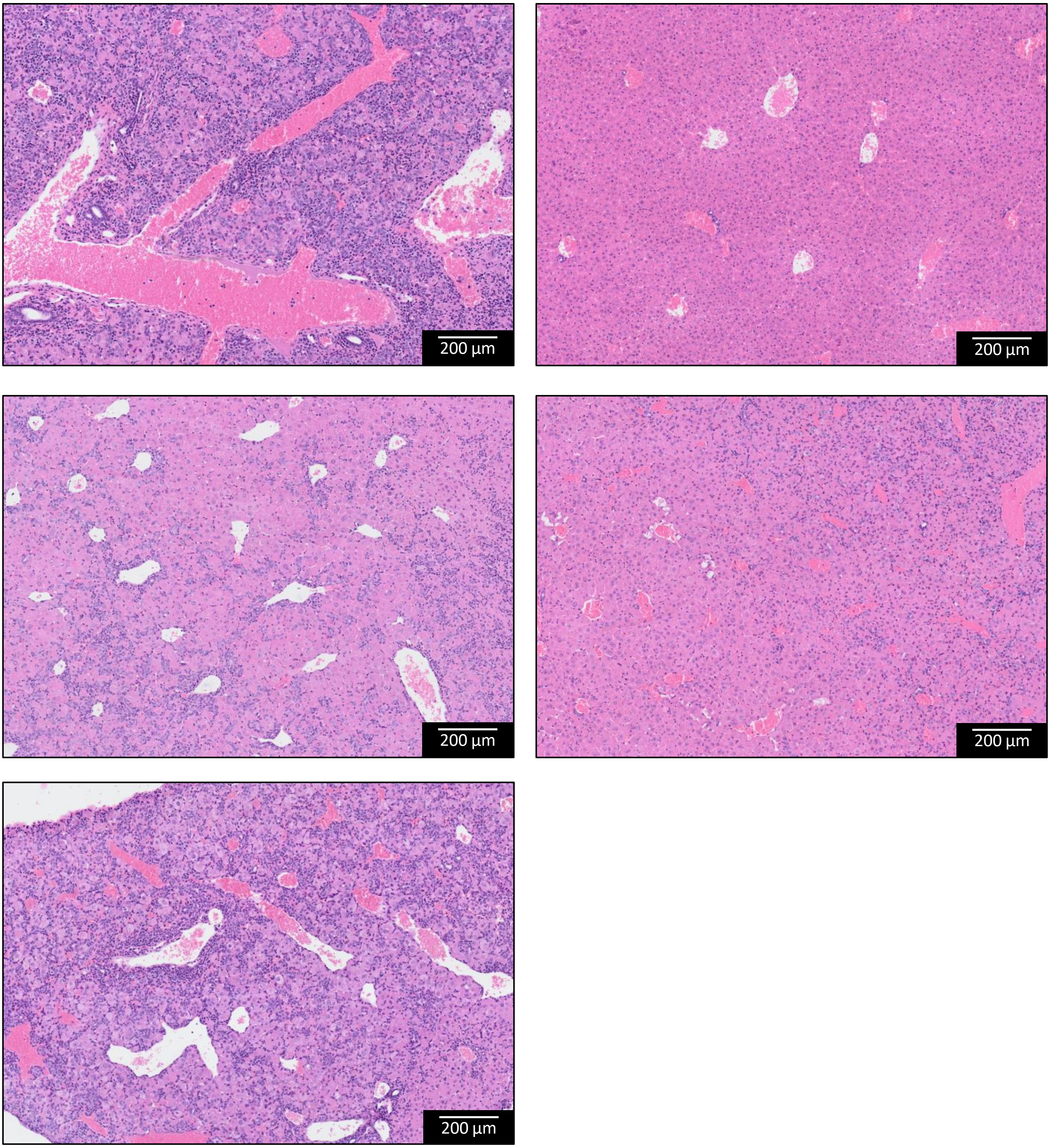

female *Slc30a10*$^{-/-}$

Figure S8

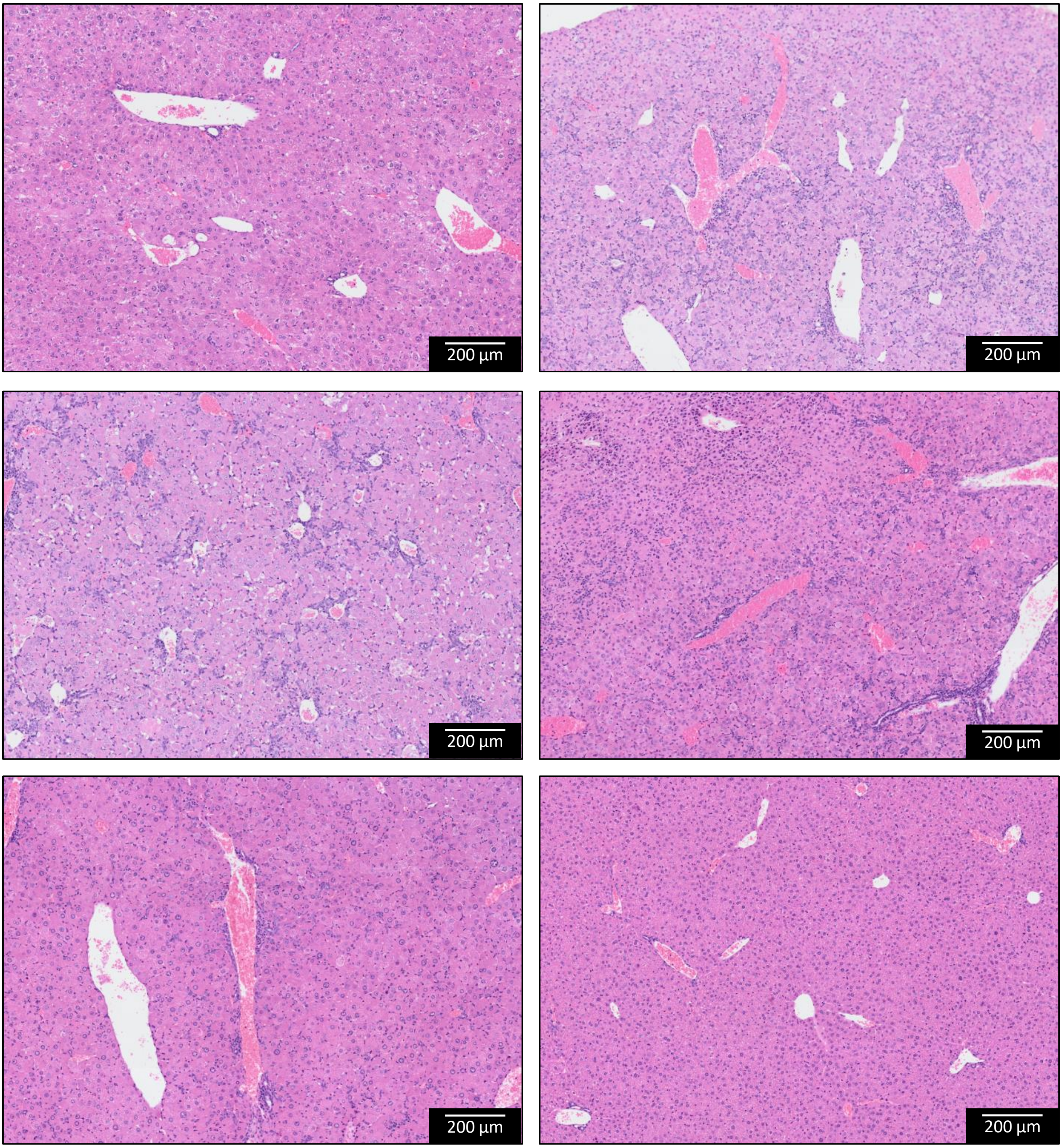

male *Slc30a10*$^{-/-}$ Figure S9

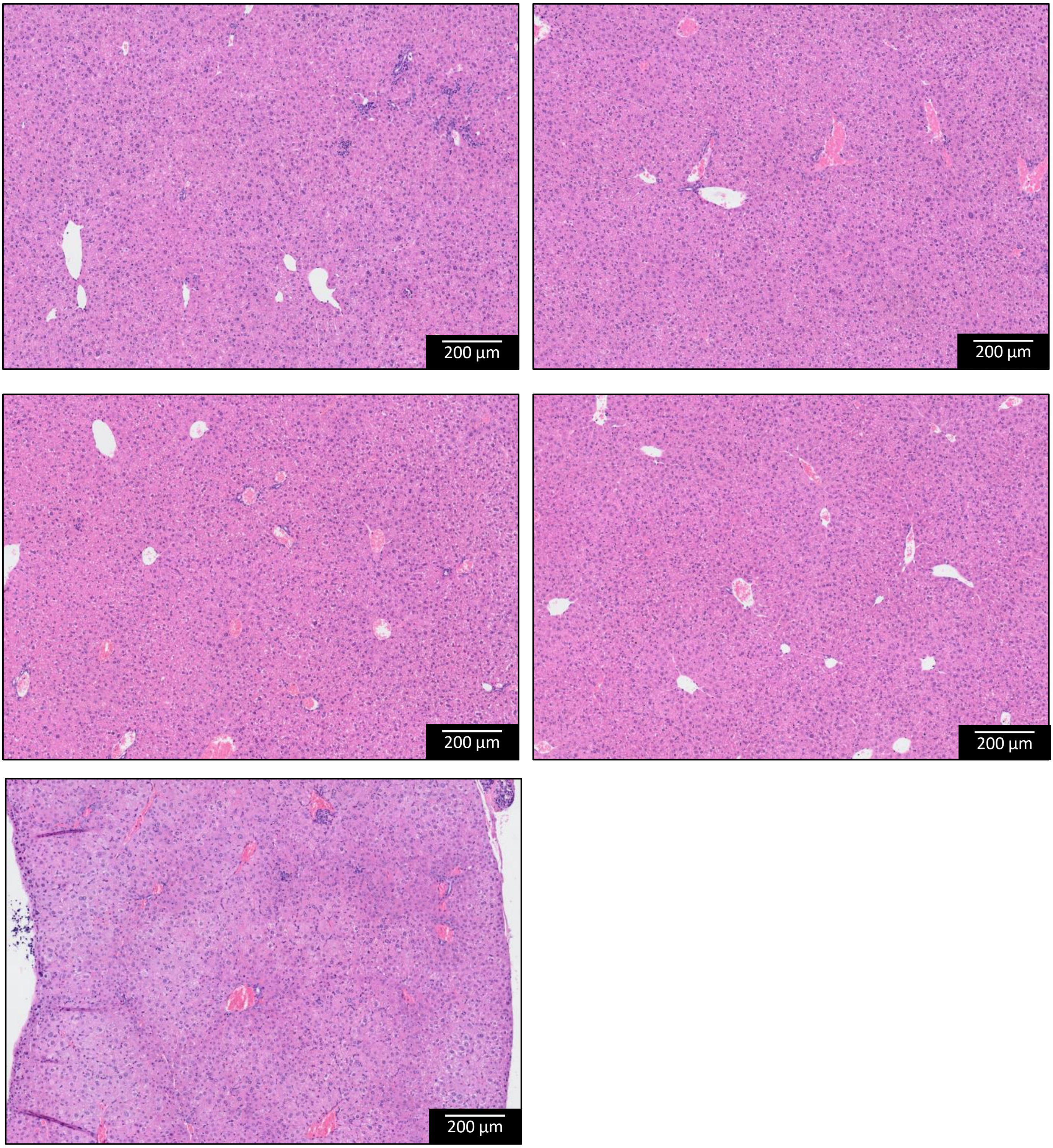

female *Slc30a10*$^{-/-}$ + T95 AAV

Figure S10

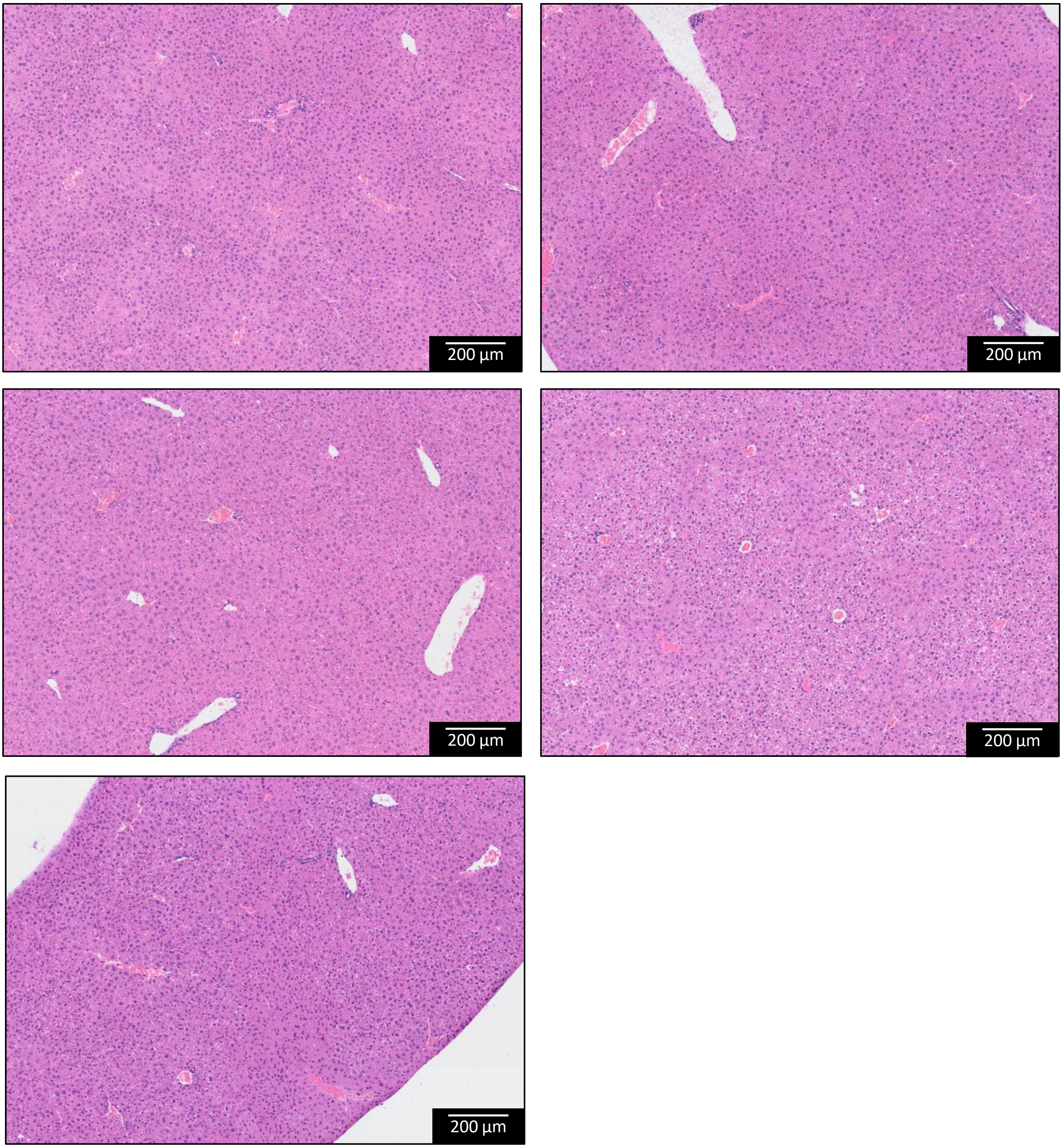

male *Slc30a10*$^{-/-}$ + T95 AAV

Figure S11

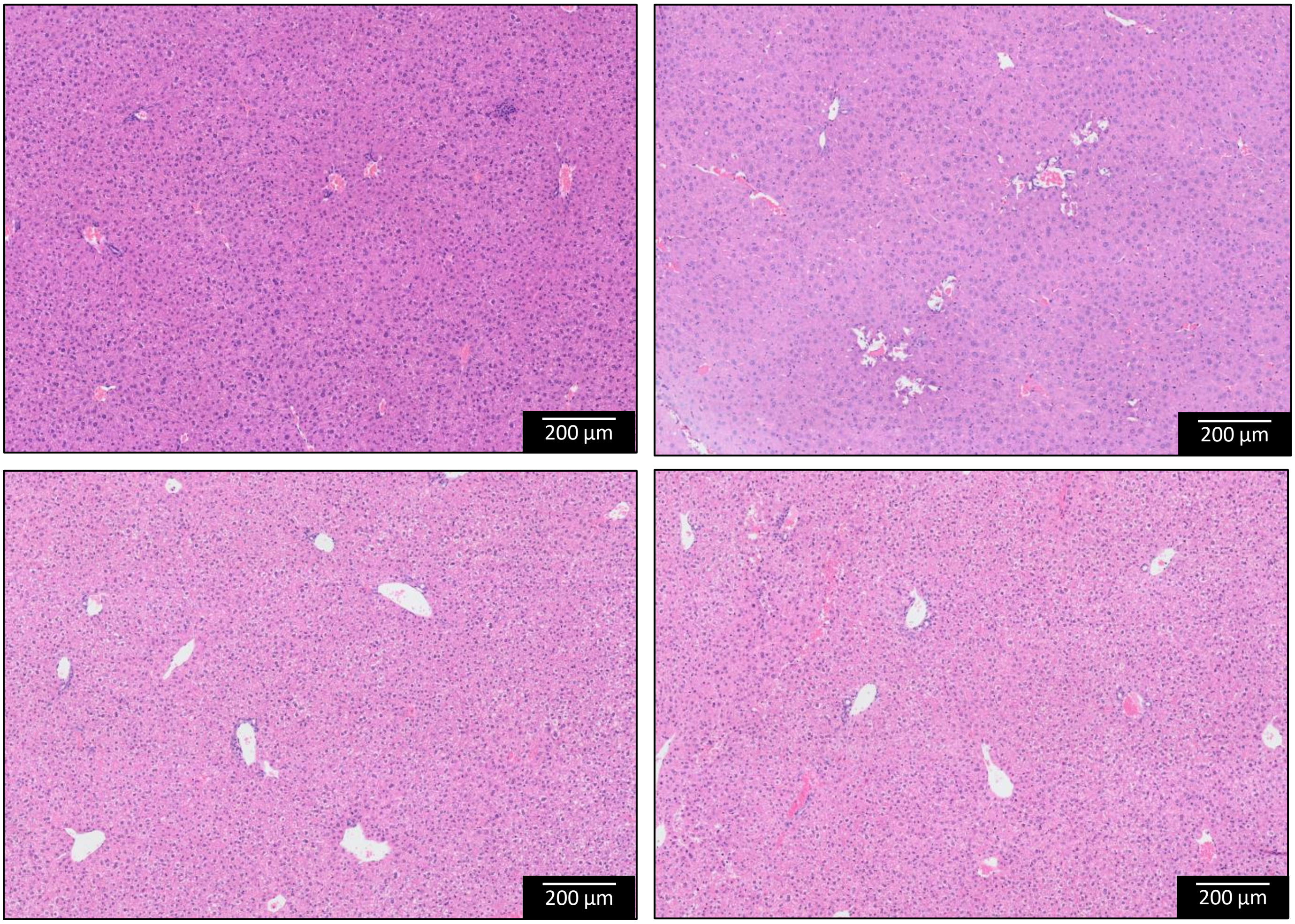

Female *Slc30a10*$^{-/-}$ + I95 AAV

Figure S12

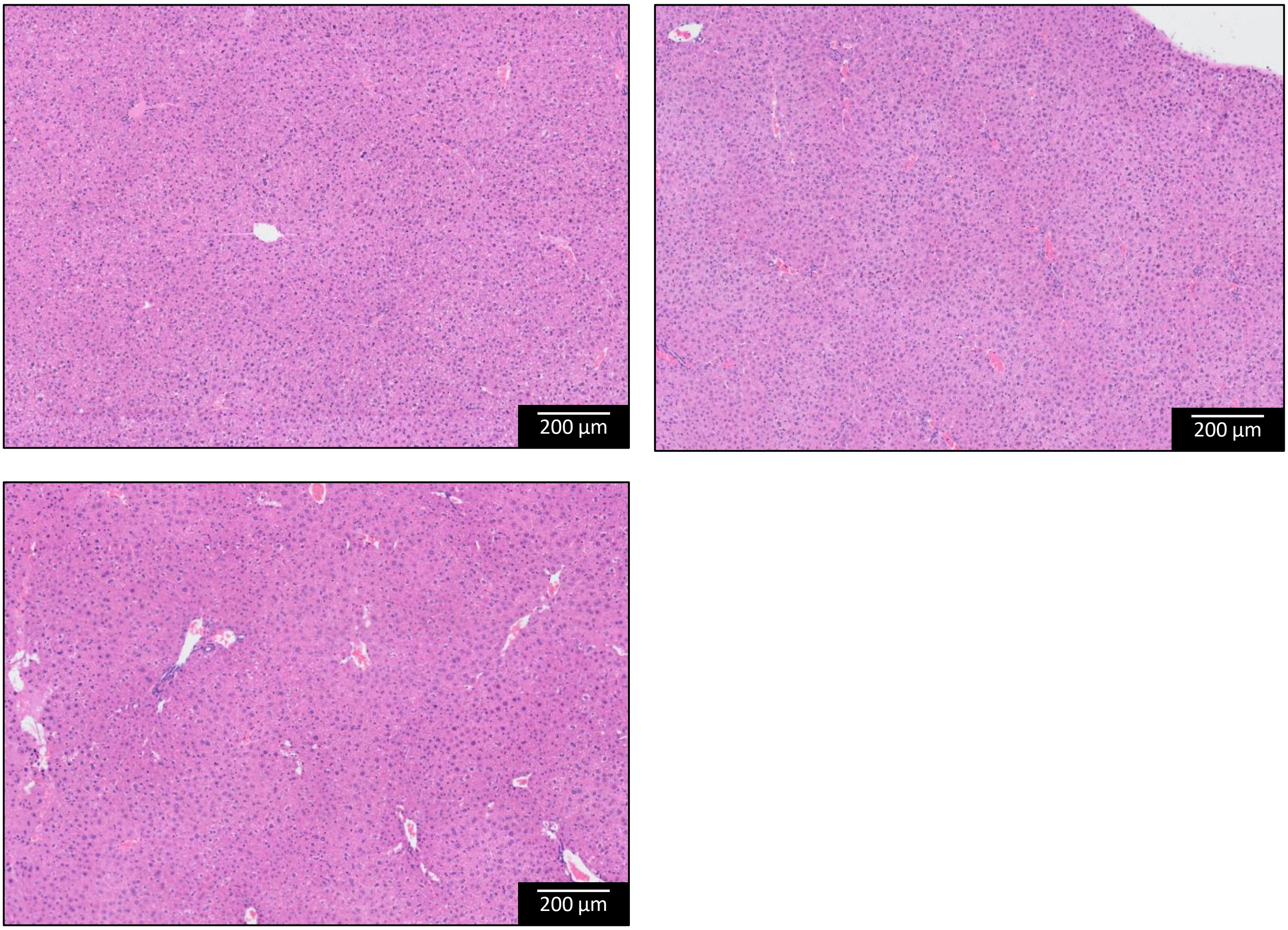

male *Slc30a10*$^{-/-}$ + I95 AAV Figure S13
